# Advances and Applications of Oxidized van der Waals Transition Metal Dichalcogenides

**DOI:** 10.1002/advs.202407175

**Published:** 2024-09-23

**Authors:** Brian S. Y. Kim, Tien Dat Ngo, Yasir Hassan, Sang Hoon Chae, Soon‐Gil Yoon, Min Sup Choi

**Affiliations:** ^1^ Department of Materials Science and Engineering University of Arizona Tucson AZ 85721 USA; ^2^ Department of Physics University of Arizona Tucson AZ 85721 USA; ^3^ imec Remisebosweg 1 Leuven 3001 Belgium; ^4^ Department of Materials Science and Engineering Chungnam National University Daejeon 34134 Republic of Korea; ^5^ School of Electrical and Electronic Engineering Nanyang Technological University Singapore 639798 Singapore; ^6^ School of Materials Science and Engineering Nanyang Technological University Singapore 639798 Singapore

**Keywords:** 2D materials, surface oxidation, transition metal dichalcogenides, van der Waals heterostructures

## Abstract

The surface oxidation of 2D transition metal dichalcogenides (TMDs) has recently gained tremendous technological and fundamental interest owing to the multi‐functional properties that the surface oxidized layer opens up. In particular, when integrated into other 2D materials in the form of van der Waals heterostructures, oxidized TMDs enable designer properties, including novel electronic states, engineered light‐matter interactions, and exceptional‐point singularities, among many others. Here, the evolving landscapes of the state‐of‐the‐art surface engineering technologies that enable controlled oxidation of TMDs down to the monolayer‐by‐monolayer limit are reviewed. Next, the use of oxidized TMDs in van der Waals heterostructures for different electronic and photonic device platforms, materials growth processes, engineering concepts, and synthesizing new condensed matter phenomena is discussed. Finally, challenges and outlook for future impact of oxidized TMDs in driving rapid advancements across various application fronts is discussed.

## Graphical abstract

This review covers recent progress in oxidized 2D transition metal dichalcogenides and their integration into other 2D materials in the form of van der Waals heterostructures. This review focuses on the new designer properties and functionalities that emerge through this integration, along with a critical outlook on promising next steps that will guide the focus and help advance the field.

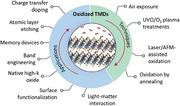

## 1 Introduction

The heterogeneous integration of dissimilar materials has repeatedly had a transformative impact on exploring new emergent phenomena and realizing the next generation of electronic and photonic devices with new functionalities. This concept was initially explored extensively in epitaxial thin film heterostructures.^[^ ^1^, ^2^ ^]^ However, the stringent growth‐matching conditions required to realize high‐quality epitaxial heterostructures and thin films drastically limit the choice and combination of available materials. This concept was recently extended to 2D heterostructures.^[^ ^3^, ^4^, ^5^, ^6^ ^]^ The latter approach has proven particularly powerful, as it does not require consideration of the structural or chemical compatibility of the constituent materials, enabling the straightforward assembly of various 2D materials with layer‐specific attributes. The presence of a van der Waals (vdW) gap and dangling‐bond‐free surface in 2D materials provides an additional competitive advantage by enabling the straightforward preparation and layer‐by‐layer assembly of the desired heterostructures.^[^ ^7^, ^8^, ^9^, ^10^ ^]^

More recently, the heterogeneous integration of hybrid heterostructures that involve more than one class of material systems has rapidly gained interest as it can diversify new functionalities and applications.^[^ ^11^, ^12^, ^13^, ^14^ ^]^ This review particularly focuses on the integration of 2D materials with transition‐metal oxides (TMOs), an extensively studied class of materials displaying diverse emergent phenomena resulting from the unique strong correlation of electronic degrees of freedom in *d* electron systems.^[^ ^15^ ^]^ In applications, oxides can also serve as a robust encapsulation layer for improved device stability, a high‐k dielectric for significantly scaled ultra‐low‐power devices, and a versatile quantum and nonlinear optical platform.^[^ ^16^, ^17^ ^]^ This review focuses on using oxidized 2D materials and their heterostructures to enable an entirely dry stacking approach for realizing pristine and atomically sharp 2D/oxide interfaces.^[^ ^18^ ^]^ For example, the clean interface initially formed during the vdW stacking step is naturally preserved throughout the post‐oxidation step.^[^ ^19^ ^]^ The post‐oxidation techniques are technologically appealing because many of these techniques are CMOS‐compatible, operate near room temperature, are facile and economical to operate, and offer fast‐throughput verification of new device structures.

**Figure 1:**
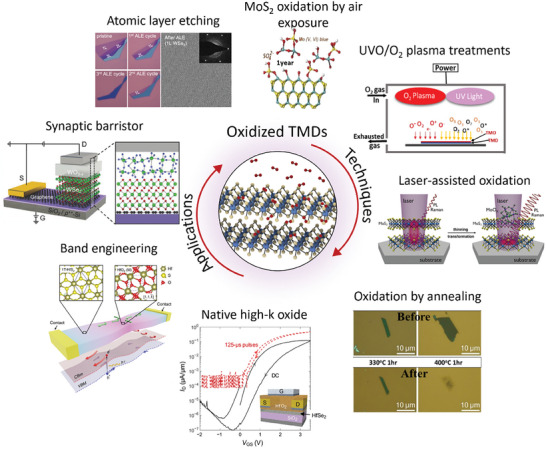
An overview of oxidation techniques for TMDs and their applications. Image for “MoS_2_ oxidation by air exposure”: Reproduced with permission.^[^ ^21^ ^]^ Copyright 2019, American Chemical Society. Image for “Oxidation by annealing”: Reproduced with permission.^[^ ^22^ ^]^ Copyright 2013, Wiley‐VCH. Image for “Laser‐assisted oxidation”: Reproduced with permission.^[^ ^23^ ^]^ Copyright 2020, Wiley‐VCH. Image for “Atomic layer etching”: Reproduced with permission.^[^ ^24^ ^]^ Copyright 2021, American Chemistry Society. Image for “Synaptic barrister”: Reproduced with permission.^[^ ^25^ ^]^ Copyright 2018, Wiley‐VCH. Image for “Band engineering”: Reproduced under the terms of the CC‐BY 4.0 license.^[^ ^26^ ^]^ Copyright 2018, The Authors, Published by Springer Nature. Image for “Native high‐k oxide”: Reproduced under the terms of the CC‐BY‐NC 4.0 license.^[^ ^27^ ^]^ Copyright 2017, The Authors, Published by American Association for the Advancement of Science.

2D transition‐metal dichalcogenides (TMDs) are promising candidates for post‐oxidation schemes because both TMDs and TMOs share the same transition‐metal ions. In this review, we first broadly cover the recent progress in various post‐oxidation techniques and their oxidation mechanism developed for oxidizing TMDs into oxides down to the layer‐by‐layer resolution limit. We also discuss approaches that enable the patterned oxidation of TMDs to create new types of nanostructures with interesting functionalities. ref. [20] provides a more focused review of passivation techniques using oxidized TMDs. Finally, we provide an overview of the wide variety of applications of oxidized TMDs, ranging from electronics to nanophotonics, and conclude with the outlook of oxidized TMDs (**Figure** **1**).

## 2 Mechanism of Oxidation

The oxidation of the basal plane surface of 2D TMDs is initiated by using appropriate oxidizing agents combined with thermal energy, light illumination, or electric field that provide sufficient energy to overcome the oxidation barrier. The oxidation process involves the physisorption of the oxygen molecule on top of the chalcogenide atoms,^[^ ^28^, ^29^ ^]^ followed by chemical reaction between chalcogenide atoms and physisorbed oxygen molecules that drives oxidation of the basal plane surface of TMDs.^[^ ^30^, ^31^, ^32^ ^]^ Chalcogenide atoms can be removed during this chemical reaction in the form of volatile gas phase of XO_2_ (X is chalcogenide atom).^[^ ^31^, ^32^ ^]^ The oxidation of TMDs is also strongly dependent on the oxidation strength of oxidizing agents. The strength of these oxidizing agents scales with their electrochemical potential – the more positive the potential, the stronger the oxidizing agent. For example, the electrochemical potential of oxygen at room temperature is 1.23 eV, whereas that for ozone and singlet oxygen are significantly higher at 2.07 and 2.42 eV, respectively.^[^ ^33^, ^34^ ^]^ Oxygen plasma can serve as even more potent and denser oxidizing agents, for which oxidation strength can be modified on demand by finely tuning the parameters used to generate the plasma.

A number of different techniques are utilized to oxidize TMDs. For example, air exposure or annealing uses a range of thermal energy together with oxygen in ambient air to drive oxidation.^[^ ^21^, ^22^ ^]^ On the other hand, oxygen plasma uses high‐power radio waves to ionize oxygen molecules into an energetic plasma, which can directly impact and oxidize the surface of TMDs.^[^ ^35^ ^]^ Another commonly used oxidation technique is exposing TMD surface with ultraviolet (UV)‐ozone environment.^[^ ^19^, ^24^ ^]^ Here, UV is typically absorbed by ozone and does not reach the TMD surface, so the main oxidizing agent is ozone molecule. But UV light also indirectly facilitates oxidation by dissociating ozone into a singlet atomic oxygen, which also strongly oxidizes the surface of TMDs.^[^ ^36^ ^]^ Other examples include oxidation by illuminating intense laser beams to facilitate chemical reaction with ambient air or by using an atomic force microscopy (AFM).^[^ ^23^ ^]^ The latter method involves the use of H_2_O as an oxidizing agent in the form of a water bridge formed between the AFM tip and the target sample.^[^ ^37^ ^]^ This water bridge together with an applied electric field across the AFM tip and sample locally drive chemical reaction under the AFM tip. A detailed discussion of common oxidation techniques used for oxidizing TMDs will further be discussed in the next section.

The oxidation behavior of 2D TMDs varies with their kinetic energy barriers to oxygen dissociation, which strongly depends on the type of chalcogen element. Among group VIB TMDs, those containing sulfur (S), such as MoS_2_ and WS_2_, are recognized as the most air‐stable materials. These materials possess relatively high kinetic energy barriers to oxygen dissociation, with the energy barrier ranging from 1.59 eV for MoS_2_ to 1.29 eV for WS_2_.^[^ ^28^, ^29^ ^]^ Oxidation in these materials predominantly occurs from the edges, which are more susceptible to oxidation than the basal planes as the activation energy is lower near edges due to lattice mismatch.^[^ ^29^ ^]^ In contrast, TMDs containing selenium (Se) or tellurium (Te) exhibit lower resistance to oxidation. For instance, the kinetic energy barrier for oxygen dissociation decreases from 1.59 eV in MoS_2_ to 0.92 eV in MoSe_2_.^[^ ^28^ ^]^ Despite this reduced kinetic energy barrier, Se‐ and Te‐based TMDs also experience oxidation primarily initiating at the edges rather than on the basal planes.^[^ ^28^ ^]^ For group IVB TMDs, such as TiX_2_, HfX_2_, and ZrX_2_, or group VB TMDs, such as NbX_2_ and TaX_2_ (where X = S, Se, Te), the kinetic energy barrier for oxidation is typically very low. Thus, these materials can undergo rapid oxidation even upon brief exposure to air at ambient conditions, leading to the formation of TMO on their surface.^[^ ^38^ ^]^ It was also shown that chalcogenide elements can be produced as byproducts of the oxidation process, which can in turn intercalate into the vdW gap of underlying TMD layers. This latter process enlarges the vdW gap, through which oxygen can penetrate and induce oxidation in the underlying TMD layers. This unique sequence of oxidation–selenium‐intercalation mechanism facilitates the oxidation of multiple layers over an extended period of time.^[^ ^39^ ^]^

## 3 Oxidation Techniques

### 3.1 Air Exposure

**Figure 2:**
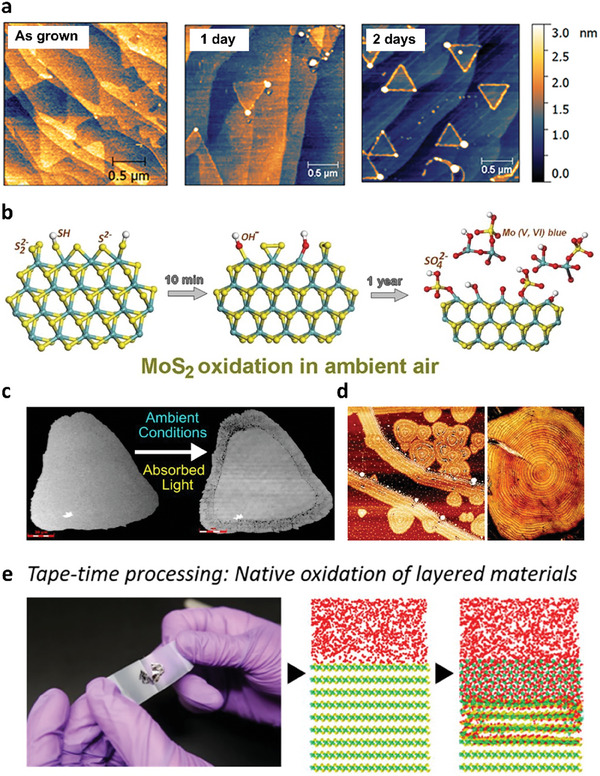
a) AFM images of WS_2_ on epitaxial graphene with different aging times. Reproduced with permission.^[^ ^45^ ^]^ Copyright 2020, American Chemical Society. b) Schematic representation of MoS_2_ oxidation steps. Reproduced with permission.^[^ ^21^ ^]^ Copyright 2019, American Chemical Society. c) Photoinduced oxidation of monolayer WS_2_ under ambient conditions. Reproduced with permission.^[^ ^49^ ^]^ Copyright 2019, American Chemical Society. d) Tree‐ring oxidation of WSe_2_. Reproduced with permission.^[^ ^53^ ^]^ Copyright 2021, Royal Society of Chemistry. e) Atomistic mechanisms of ZrS_x_Se_2‐x_ oxidation under ambient conditions. Reproduced with permission.^[^ ^58^ ^]^ Copyright 2020, American Chemical Society.

The atomically thin nature of 2D materials intrinsically results in high susceptibility to environments and low stability in air.^[^ ^40^ ^]^ Among group VIB TMDs, it is generally agreed that Te‐based materials (MoTe_2_ and WTe_2_) are air‐sensitive while S‐ or Se‐based materials (MoS_2_, MoSe_2_, WSe_2_, and WS_2_) are relatively stable in air. However, recent studies revealed that field‐effect transistors (FETs) fabricated with the relatively air‐stable WSe_2_ can also suffer from a significant reduction in the drain current when metallization is performed under ambient conditions relative to those performed in an inert environment.^[^ ^41^ ^]^ Moreover, a distinctive hysteresis is often observed when 2D devices are measured in ambient conditions due to charge trapping.^[^ ^42^ ^]^ These observations suggest that an even small amount of trapped air or water molecules at metal/2D semiconductor interfaces can degrade the performance of 2D devices.^[^ ^43^, ^44^ ^]^ Further studies show that intrinsic defect sites, such as edge defects present in 2D materials, promote the adsorption of oxygen or other impurities on the 2D surface, as shown in **Figure** **2**a.^[^ ^28^, ^32^, ^45^, ^46^ ^]^ Because of the unfavorable stability of 2D TMDs in air, several studies have investigated the oxidation of such materials by simple air exposure.^[^ ^47^ ^]^

Among the TMDs, MoS_2_ is one of the most promising candidates for 2D TMDs for applications in electronic devices owing to its stability in air. Nevertheless, Afanasiev et al. found that the edge of the MoS_2_ flake can be rapidly oxidized after just 10 mins of air exposure.^[^ ^21^ ^]^ Figure 2b illustrates the oxidation steps of MoS_2_. The edge sulfur species formed hydroxyl (‐OH) groups upon short‐term air exposure, followed by the detachment of oligomeric molybdenum species upon prolonged exposure. This oxidation process was accelerated under humid conditions, leading to deeper oxidation without producing a protective passivation layer with the remaining sulfide atoms. Moreover, the existence of a trace amount of MoO_x_ in the as‐grown CVD MoS_2_ activated the oxidation process even at a moderate temperature of 100 °C under ambient pressure.^[^ ^48^ ^]^ Thus, the intrinsic defect density was crucial for controlling the ambient oxidation of MoS_2_.

For WS_2_, without light activation and elevated temperatures, the flakes had no signs of oxidation even after 10 months left in an ambient environment.^[^ ^49^, ^50^ ^]^ However, they underwent a strong photo‐oxidation process after 7 days under illumination with 532 or 660 nm lasers, as shown in Figure 2c, which is visible in the laser scanning confocal micrographs. Most of the oxidized areas originated from the flake edges or sulfur vacancy‐rich areas. Chang et al. found that this photoinduced oxidation mechanism is mainly due to formation of O^2−^ radicals.^[^ ^51^ ^]^ Furthermore, CVD‐grown WS_2_ on epitaxial graphene showed an accelerated oxidation compared to that on a sapphire substrate owing to enhanced adsorption of gas species in ambient environments.^[^ ^45^ ^]^ Similarly, for CVD‐grown WSe_2_, the 1D WSe_2_ edge was immediately oxidized after air exposure, forming a metallic tungsten oxide.^[^ ^52^ ^]^ The air oxidation process can also be initiated at defect sites of the exfoliated WSe_2_ flakes, such as edge‐ and point‐defects, resulting in unique tree‐ring/line shaped oxidation, as shown in Figure 2d.^[^ ^53^ ^]^ Another group reported that chemisorption of oxygen species with prolonged ambient exposure leads to a permanent shift in the Fermi energy due to oxidation.^[^ ^54^ ^]^

For relatively unstable TMDs such as MoTe_2_ and WTe_2_, an ultrathin amorphous layer MoO_3_‐TeO_2_ was immediately formed when CVD or MBE‐grown MoTe_2_ was exposed to ambient environments, which strongly affected the electronic properties.^[^ ^55^, ^56^, ^57^ ^]^ Among group IVB TMDs (ZrSe_2_, ZrS_2_, HfS_2_, and HfSe_2_), HfSe_2_ readily formed Se‐rich blisters due to oxidation within one day of air exposure.^[^ ^38^ ^]^ Jo et al. reported that increasing the Se content in ZrS_x_Se_2‐x_ increased the oxidation rate in an ambient atmosphere, as depicted in Figure 2e.^[^ ^58^ ^]^ A relatively thick native oxide layer, which can be used as high‐k dielectrics, was also formed within several days by exposing HfSe_2_, ZrSe_2_, and HfS_2_ to air.^[^ ^27^ ^]^ However, oxidation of HfSe_2_ in air creates a relatively low‐quality HfO_x_ layer with a bumpy surface due to moisture and oxygen in air.^[^ ^59^ ^]^ The relatively small band gap (≈2 eV) of the native oxide is also a disadvantage for electronic devices.^[^ ^60^ ^]^

TiSe_2_, NbSe_2_, and TaS_2_ are members of air‐sensitive 2D TMDs that exhibit superconductivity and charge density wave (CDW) ordering above superconducting transition temperatures. Interestingly, Sun et al. showed that an air exposure of TiSe_2_ oxidizes its surface and suppresses the characteristic CDW ordering.^[^ ^39^ ^]^ The oxidation process can be explained in terms of a sequence of the following two steps. Initially, air exposure renders protruded edges that eventually grow laterally and form a homogenously oxidized layer on the surface of TiSe_2_. The air‐exposed oxidation produces elemental selenium atoms as byproducts, which in turn intercalate into the vdW gap in between the underlying TiSe_2_ layers. The intercalation of Se atoms increases the vdW gap and allows oxygen to diffuse and oxidize deep TiSe_2_ layers through the edges of the enlarged vdW gap. This oxidation–selenium‐intercalation mechanism can also describe the oxidation process of air‐exposed NbSe_2_ and TaS_2_.

### 3.2 Oxygen plasma Treatments

**Figure 3:**
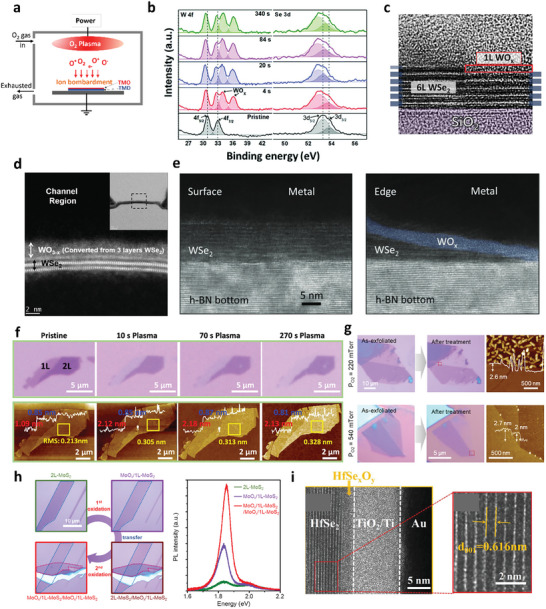
a) Schematic representation of the oxidation of TMDs by oxygen plasma treatment. b) XPS spectra for the oxidized WSe_2_ at different oxidation times. Reproduced with permission.^[^ ^66^ ^]^ Copyright 2019, Royal Society of Chemistry. c) Cross‐sectional TEM image of a 6L WSe_2_ with and without O_2_ plasma treatment. Reproduced under the terms of the CC‐BY 4.0 license.^[^ ^70^ ^]^ Copyright 2021, The Authors, Published by IOP Publishing. d) Cross‐sectional TEM image of a WSe_2_ flake, indicating that approximately three layers of WSe_2_ convert into WO_x_ by high‐temperature oxygen plasma treatment. Reproduced with permission.^[^ ^67^ ^]^ Copyright 2020, Wiley‐VCH. e) Cross‐sectional TEM images of WSe_2_ with surface and edge contacts. Reproduced with permission.^[^ ^71^ ^]^ Copyright 2022, Wiley‐VCH. f) Optical micrographs and corresponding AFM images of a MoTe_2_ flake with both monolayer and bilayer parts before and after O_2_ plasma treatments. Reproduced with permission.^[^ ^78^ ^]^ Copyright 2020, American Chemical Society. g) Optical and AFM images of MoS_2_ before and after oxygen plasma treatment with different O_2_ pressures. h) Formation of multiple MoS_2_/MoO_x_ heterostructures and the corresponding PL intensities. Images in g) and h) reproduced with permission.^[^ ^79^ ^]^ Copyright 2021, American Chemical Society. i) Cross‐sectional TEM image of the oxidized HfSe_2_ device. Reproduced under the terms of the CC‐BY 4.0 license.^[^ ^80^ ^]^ Copyright 2021, The Authors, Published by Wiley‐VCH.

Plasma treatment is a common surface‐functionalization method for 2D materials. The main operating principle of plasma treatments lies in the partially ionized gas consisting of electrons, ionized molecules, excited molecules, and radicals bombarding the sample surface, as illustrated in **Figure** **3**a. Plasma treatment utilizing oxygen gas is known to be an effective approach for oxidizing 2D TMDs.^[^ ^61^, ^62^, ^63^, ^64^, ^65^ ^]^ Unlike air exposure, which usually requires a long period of exposure with the appearance of moisture to form an oxide layer, the reactive components in oxygen plasma stimulate the oxidation of TMDs, forming an oxide layer within a few seconds under high‐vacuum conditions (Figure 3b).^[^ ^66^ ^]^

Due to their high reaction rate, oxygen plasma treatments are commonly employed on WSe_2_ because the self‐limiting nature of WO_x_ can prevent further oxidation of the underlying layers.^[^ ^67^, ^68^ ^]^ Remote oxygen plasma treatment was found to provide self‐limiting oxidation due to high chemical reactivity without physical bombardment.^[^ ^69^ ^]^ Moon et al. demonstrated that only the topmost single layer of WSe_2_ is oxidized, while the underlying layers of WSe_2_ remain intact after oxygen inductively coupled plasma (ICP) treatment at room temperature, as evidenced from the cross‐sectional TEM images in Figure 3c.^[^ ^66^, ^67^, ^68^, ^69^, ^70^ ^]^ The self‐limiting nature of WSe_2_ cannot be preserved when oxygen plasma treatment is performed at elevated temperatures. For example, Pang et al. reported that plasma treatments at 250 °C can oxidize the top three layers of WSe_2_ into WO_x_ using a plasma‐enhanced CVD (PECVD) system (Figure 3d).^[^ ^67^ ^]^ This increase in the oxidation depth can be understood as a reduction in the activation barrier for oxygen penetration through the preformed oxidized layers, leading to the subsequent oxidation of the underlying WSe_2_. High‐temperature plasma treatment was also found to oxidize WSe_2_ underneath the protective metal electrodes via the horizontal penetration of oxygen species. This suggests that the temperature is an effective measure for precisely controlling the thickness of the oxide layer. Further studies on the relationship between the oxidation depth and temperature are required to understand the atomic dynamics of oxygen penetration at different temperatures.

In contrast, a thick nonstoichiometric WO_x_ layer was generated at the WSe_2_ edges after the etching process to form 1D edge contacts via SF_6_/O_2_ plasma treatments (Figure 3e), which enabled the formation of a p‐n junction.^[^ ^71^, ^72^ ^]^ The WO_x_ layer was found to induce strong Fermi level pinning (FLP) owing to its strong p‐doping effects. Furthermore, such WO_x_ formation and defect creation via plasma treatment enhanced the photocurrent 150‐fold, which can be implemented in high‐performance optoelectronic applications.^[^ ^73^ ^]^

In Mo‐based TMDs such as MoS_2_, it has been reported that only the top TMD layer transforms into the MoO_3_ layer, which prevents further transformation of the lower MoS_2_ layer from oxidation.^[^ ^74^, ^75^ ^]^ This self‐limiting process enables the cleaning of polymer residues during the lithographic process, providing a clean interface between the metal and MoO_x_.^[^ ^76^, ^77^ ^]^ However, oxygen ions can diffuse into the underlying MoS_2_ and induce strain in the crystal structure, as indicated by the relative shift in the A_1g_ Raman peak. Moreover, the uniformity of oxidation strongly depends on the specific oxidation technique used. For example, plasma treatment at 200 °C resulted in a relatively uniform MoO_x_ layer on the bulk MoS_2_ surface. In contrast, the thermal annealing of MoS_2_ in air induced an inhomogeneous MoO_x_ layer consisting of voids and wrinkles.^[^ ^61^ ^]^

Liu et al. performed oxygen plasma treatment on bilayer MoTe_2_, which is a relatively unstable 2D TMD, to realize layer‐by‐layer oxidation.^[^ ^78^ ^]^ This study showed that only the top layer of the bilayer MoTe_2_ was converted into MoO_x_, while the remaining bottom layer was preserved even with further oxygen plasma treatment, as demonstrated in the optical and AFM images in Figure 3f. Kang et al. developed an optimal oxygen plasma condition with high‐pressure (540 mTorr) during treatment, avoiding severe bombardment of ions on the MoS_2_ surface; defect‐free monolayer MoS_2_ was therefore obtained by oxidizing bilayer MoS_2_.^[^ ^79^ ^]^ The ultraclean surface of oxidized MoS_2_ indicates the high quality of oxidation, as shown in Figure 3g. Thus, the top single layer of WO_x_ or MoO_x_ formed by oxygen plasma treatment can be an effective passivation layer. The passivation ability can significantly enhance the PL intensity of MoS_2_ in the integrated MoS_2_/MoO_x_ heterostructures because the stacked MoS_2_ layers can be decoupled by the formed MoO_x_ layers (Figure 3h).

For group IV TMDs such as HfSe_2_, the utilization of oxygen plasma enables the formation of a high‐quality oxide layer, in contrast to the uneven oxide surface resulting from air exposure during the oxidation process.^[^ ^59^, ^80^ ^]^ Additionally, the depth of oxidation in HfS_2_ can be effectively controlled by adjusting the duration of the oxygen plasma treatment.^[^ ^81^ ^]^ In general, unlike group VI TMDs, the oxidation process of group IV TMDs through oxygen plasma treatments does not exhibit a self‐limiting nature, as evidenced by the formation of a relatively thick oxide layer in the cross‐sectional TEM images in Figure 3i.^[^ ^80^ ^]^

### 3.3 UV‐Ozone Oxidation

**Figure 4:**
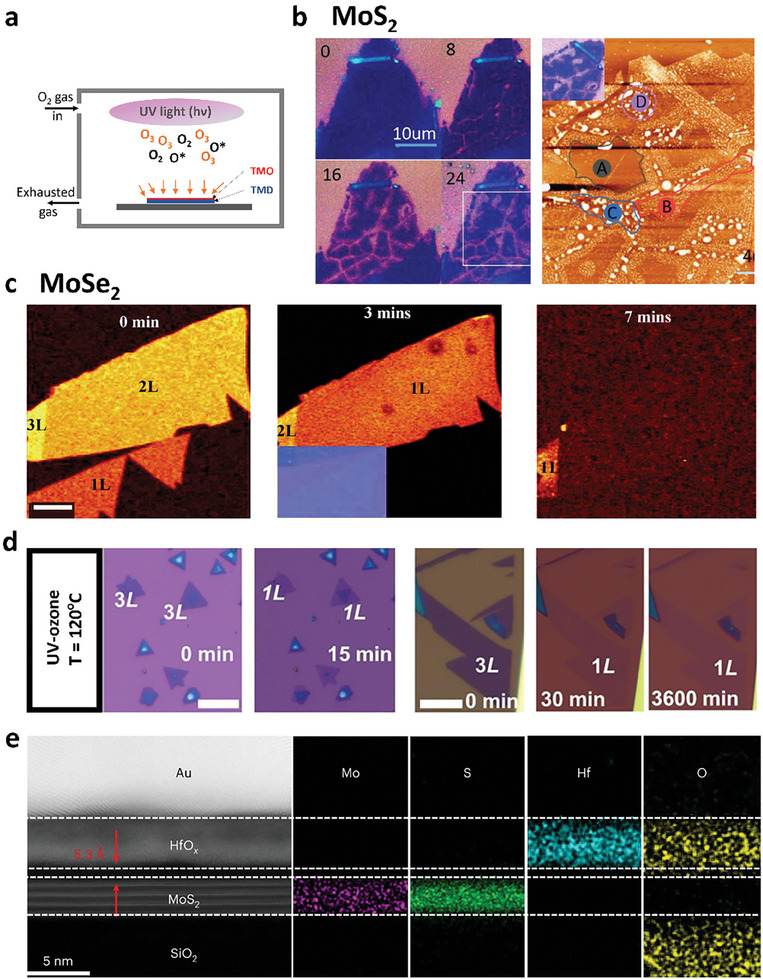
a) Schematic of oxidation process for TMDs by UV‐ozone treatment. b) Optical images of 3L MoS_2_ with 0‐, 8‐, 12‐, and 24‐min treatments and AFM image after 24 min treatment. Reproduced under the terms of the CC‐BY‐NC‐ND 4.0 license.^[^ ^84^ ^]^ Copyright 2020, The Authors, Published by Elsevier B.V. c) Raman mapping of MoSe_2_ upon sequent UV‐ozone treatments for 0, 3, and 7 min. Reproduced with permission.^[^ ^85^ ^]^ Copyright 2019, Royal Society of Chemistry. d) Optical images of MoS_2_ flakes before and after UV‐ozone treatments. Reproduced with permission.^[^ ^88^ ^]^ Copyright 2022, American Chemistry Society. e) Cross‐sectional TEM image of HfO_x_ and MoS_2_ heterostructures and elemental distributions acquired by an EELS spectrometer. Reproduced with permission.^[^ ^93^ ^]^ Copyright 2022, Springer Nature.

UV‐ozone treatment is a gentle oxidation method commonly used to oxidize 2D materials using a UV cleaner. Unlike oxygen plasma treatments where high‐energy ions or radicals strongly bombard the surface of 2D TMDs, potentially damaging the layer, the reactive oxygen atoms (O*) produced during generation and decomposition of ozone under UV illumination (184.9 and 253.7 nm) play a pivotal role in gently oxidizing 2D TMDs, forming high‐quality oxide layers as illustrated in **Figure** **4**a.^[^ ^82^ ^]^ Despite the gentle oxidation process, UV‐ozone treatment at room temperature induces inhomogeneous surfaces and cracks for MoS_2_ (Figure 4b).^[^ ^83^, ^84^ ^]^ Such issues are attributed to the relatively large kinetic energy barrier for the oxygen dissociative adsorption process between the pure MoS_2_ crystal (1.59 eV) and defective MoS_2_ with sulfur vacancies (0.8 eV).^[^ ^29^ ^]^ In contrast, other 2D materials in the same group of Mo‐based TMDs (MoSe_2_ and MoTe_2_) can be homogeneously oxidized by UV‐ozone treatments, enabling layer‐by‐layer oxidation and thinning, as shown in Raman mapping images of MoSe_2_ with sequent UV‐ozone treatments (Figure 4c).^[^ ^24^, ^85^, ^86^ ^]^ However, Liang et al. reported that oxygen molecules tend to physisorb on the MoSe_2_ surface, while the oxide layer is formed on the MoTe_2_ surface with mild UV‐ozone treatments, inducing different hole doping densities.^[^ ^87^ ^]^

To overcome the drawback of UV‐ozone treatment for MoS_2_ oxidation, Alam et al. reported the wafer scalable and controllable oxidation of tri‐layer MoS_2_ by varying UV‐ozone treatment times, in which 2 (3) layers of MoS_2_ were oxidized with 15(30) min UV treatment at 120 °C, forming a uniform MoO_3_ film (Figure 4d).^[^ ^88^ ^]^ It is worth noting that unlike the self‐limiting nature observed in Mo‐based TMDs when subjected to oxygen plasma treatments, their oxidation using UV‐ozone treatment is not a self‐limiting process.^[^ ^74^, ^89^ ^]^ With room temperature treatments, the depth of oxidation can be readily controlled by adjusting the duration of the treatment.

In W‐based TMDs, WSe_2_ is oxidized by UV‐ozone treatment at room temperature, producing a topmost WO_x_ layer as a self‐limiting layer.^[^ ^24^, ^83^, ^90^ ^]^ Despite being self‐limiting, oxidized WSe_2_ layer is non‐stoichiometric with defect states manifesting as gap states near band edges as shown by Lin et al. using atomic‐scale scanning tunneling microscopy and spectroscopy.^[^ ^91^ ^]^ Unlike WSe_2_, WS_2_ is not self‐limited by the same UV‐ozone oxidation. Such differences in the W‐based TMDs can be explained by the different diffusion barrier strengths of S‐based TMDs compared to those of Se‐based TMDs.

For group IV TMDs, the oxidized HfS_2_ flakes produced by UV‐ozone treatment have a clean interface between the oxide layer and the remaining semiconducting layer, as demonstrated by the TEM image and elemental mapping in Figure 4e.^[^ ^92^, ^93^ ^]^ However, similar to the oxygen plasma treatment, oxidation is not a self‐limiting process.

### 3.4 Annealing

**Figure 5:**
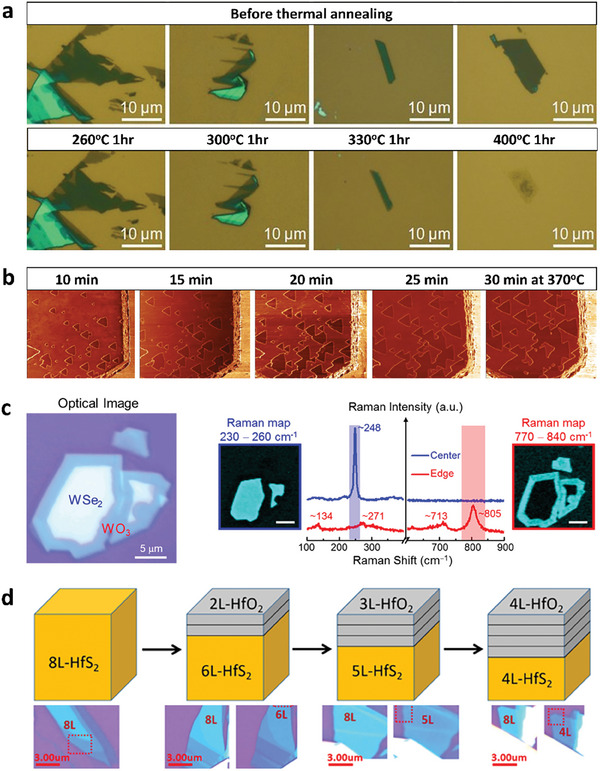
a) Optical images of MoS_2_ before and after thermal annealing at different temperatures. Reproduced with permission.^[^ ^22^ ^]^ Copyright 2013, Wiley‐VCH. b) Local force microscopic images of MoS_2_ obtained after annealing at 370 °C for 10 to 30 min, showing triangular etch pits due to oxidation. The width of all AFM images is 5 µm. Reproduced with permission.^[^ ^97^ ^]^ Copyright 2019, American Chemistry Society. c) Optical images and Raman spectra with mapping images of thermally oxidized WSe_2_. Reproduced with permission.^[^ ^103^ ^]^ Copyright 2015, American Chemistry Society. d) Schematic and optical images for the layer‐by‐layer oxidation process of HfS_2_. Reproduced with permission.^[^ ^104^ ^]^ Copyright 2020, AIP Publishing.

Investigating the formation of oxidized TMDs through annealing in an oxygen‐rich environment serves to monitor the oxidative etching process of 2D TMDs. Several studies have been conducted to elucidate the kinetics governing the heat‐induced oxidation of MoS_2_, given its significance in various industrial applications.^[^ ^94^ ^]^ The chemical interaction between MoS_2_ crystals and oxygen molecules is represented by the following reaction: MoS_2_ + 3.5O_2_ → MoO_3_ + 2SO_2_. The annealing temperature plays a pivotal role in the oxidation of MoS_2_. Below an annealing temperature of 300 °C, no discernible evidence of oxide layer formation was observed through various analytical techniques including optical microscopy (OM), AFM, scanning transmission electron microscopy (STEM), X‐ray photoelectron spectroscopy (XPS), and low‐loss electron energy loss spectroscopy (EELS) (**Figure** **5**a).^[^ ^22^, ^95^, ^96^ ^]^ Following annealing at temperatures exceeding 300 °C in ambient conditions, notable changes occurred: the color contrast of the flakes observed via OM was altered, and lateral force microscopic images revealed triangular etch pits induced by oxidation, as illustrated in Figure 5b.^[^ ^97^ ^]^ XPS spectra revealed characteristic doublets of Mo^6+^ at peak positions of ≈233 and 236 eV, and amorphous MoO_3_ was discernible in high‐resolution TEM (HRTEM) images.^[^ ^95^, ^97^ ^]^ Collectively, these findings suggest that the oxidation of MoS_2_ is initiated within the temperature range 300–400 °C.

Moreover, the formation of MoO_x_ through thermal annealing in air can lead to an inhomogeneous oxide surface.^[^ ^61^, ^98^, ^99^ ^]^ Reidy et al. demonstrated that the thermal annealing‐induced oxidation of MoS_2_ in air resulted in the simultaneous formation of MoO_x_ accompanied by MoO_x_ evaporation at ≈500 °C.^[^ ^61^ ^]^ They proposed that annealing MoS_2_ under controlled oxygen pressure partially mitigates this issue; however, pinholes were still observed on the surface of oxidized MoS_2_ using this approach. Therefore, achieving a uniform MoO_x_ layer on the surface of MoS_2_, which represents an oxidation method with an oxidation rate higher than the evaporation rate, requires further optimization.

The oxidation of MoS_2_ through annealing occurs at both the edges and basal planes of the flakes.^[^ ^100^ ^]^ The depth of oxidation in this process is contingent on both the annealing temperature and treatment duration. Intriguingly, MoS_2_ oxidation occurs even under ultra‐high vacuum (UHV) conditions when MoS_2_ is grown on oxygen‐rich substrates like SrTiO_3_.^[^ ^101^ ^]^ Utilizing the oxygen atoms present in SrTiO_3_, MoS_2_ can be oxidized at 700 °C through UHV annealing. Similarly, for WSe_2_, oxidation is observed with annealing at 400 °C in atmospheric environments.^[^ ^102^ ^]^ The thermal‐induced oxidation of WSe_2_ begins at the flake edges due to their higher oxidation reactivity relative to that at the basal plane, as evidenced by Raman mapping (Figure 5c).^[^ ^103^ ^]^ In the case of HfS_2_, the oxidation depth can be precisely manipulated by adjusting both the annealing time and the duration of exposure to ambient air.^[^ ^104^ ^]^ Unlike other group VI TMDs, the thermal‐induced oxidation of HfS_2_ can initially affect the entire surface of the flakes, leading to a layer‐by‐layer oxidation process, as depicted in Figure 5d.

The previously discussed annealing methods, which aim for the complete transformation of TMD flakes into TMOs, are impractical for applications in monolayer flakes. Therefore, a promising alternative involves the use of oxygen‐intercalated TMD flakes obtained through mild thermal annealing, which are particularly suitable for monolayer TMDs. Seo et al. demonstrated that annealing monolayer WSe_2_ at 200 °C in ambient environments enhanced p‐type conduction, indicating effective adsorption of oxygen atoms onto the surface of monolayer WSe_2_ without complete oxidation of the flakes.^[^ ^105^ ^]^ Liu et al. similarly suggested oxygen intercalation to passivate vacancies on the surface of MoTe_2_ flakes, employing low oxygen pressure annealing at 250 °C for 3 h.^[^ ^106^ ^]^

To date, most studies on thermal annealing have focused on elucidating the oxidation mechanisms of TMDs. Nevertheless, there is a pressing need for additional studies aimed at refining the ability to precisely control layer‐by‐layer oxidation, thereby facilitating the precise and comprehensive oxidation of TMDs. Furthermore, these techniques can be broadly applied to the oxidation of 2D TMDs. Nevertheless, these methods do not possess the capability to selectively oxidize specific regions of the 2D TMD surfaces, a crucial aspect for achieving a patternable doping profile and facilitating lithographic applications.^[^ ^107^, ^108^ ^]^ Several techniques have been proposed to address this limitation, such as laser‐ and AFM‐assisted methods, which enable selective oxidation without the need for lithographic patterning processes.^[^ ^109^, ^110^, ^111^, ^112^ ^]^ A related technique is using a nano‐patterned hBN mask layer that serves as a pattern transfer layer by enabling selective oxidation of 2D TMD only on the regions exposed by the holes in the hBN mask layer with desired sizes and shapes or holes.^[^ ^113^ ^]^ Using this latter method, Kim et al. demonstrated selective oxidation of WSe_2_ in desired shapes with a single‐digit nanometer lateral sharpness.

### 3.5 AFM or Laser‐Assisted Oxidation

**Figure 6:**
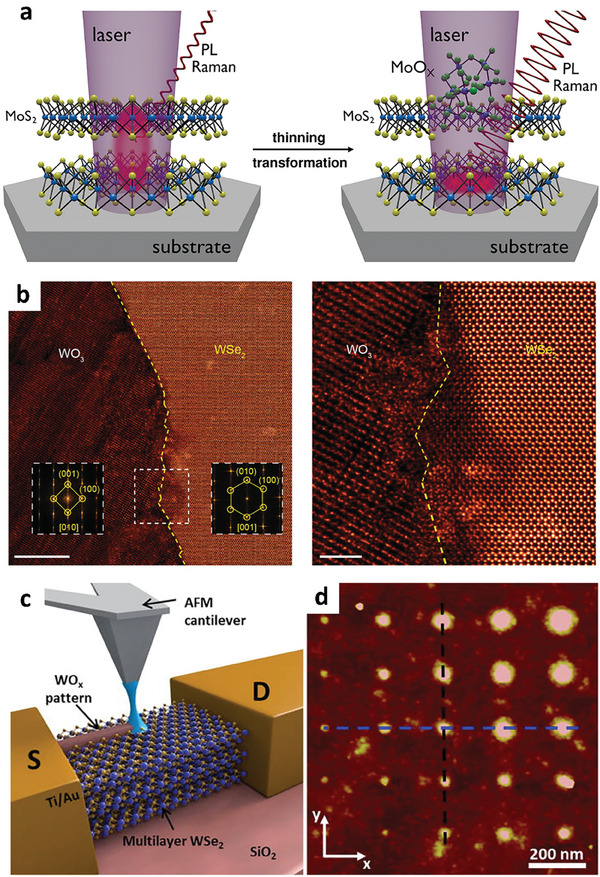
a) Illustration of laser‐induced oxidation of MoS_2_. Reproduced with permission.^[^ ^23^ ^]^ Copyright 2020, Wiley‐VCH. b) STEM images of pristine WSe_2_ in the right region and WO_3_ induced by laser irradiation in the left region. Reproduced with permission.^[^ ^118^ ^]^ Copyright 2021, American Chemistry Society. c) Schematic diagram of AFM‐assisted patterning of WO_3_ on WSe_2_. d) AFM images of an array of the patterned WO_3_ dots. Images in c) and d) reproduced under the terms of the CC‐BY 4.0 license.^[^ ^122^ ^]^ Copyright 2016, The Authors, Published by AIP Publishing.

Laser irradiation in the presence of atmospheric oxygen was utilized to selectively oxidize the surfaces of the 2D TMDs. The depth of oxidation was highly dependent on the power of the irradiating laser. For instance, Alrasheed et al. found that oxide formation occurs on the surface of MoS_2_ at a relatively high laser power, whereas the layer experiences thinning under low‐power laser illumination.^[^ ^114^ ^]^ Thus, there is a need for systematic research to identify the optimal laser power to effectively oxidize MoS_2_. This study highlights the challenge of achieving layer‐by‐layer oxidation using laser irradiation because of the concurrent thinning of MoS_2_ (**Figure** **6**a).^[^ ^23^, ^115^ ^]^ A similar thinning of MoTe_2_ flakes after laser‐assisted selective oxidation has also been reported.^[^ ^116^, ^117^ ^]^

In contrast, laser‐assisted oxidation of WSe_2_ flakes has been effectively accomplished without causing substantial thinning of the flakes under optimized conditions (Figure 6b).^[^ ^118^, ^119^ ^]^ This could be attributed to the relatively poor stability of MoO_x_ in comparison to WO_x_.^[^ ^120^ ^]^ Moreover, intense laser‐assisted oxidation leads to the formation of volatile substances such as SO_2_ and MoO_3_, which contribute to the thinning of Mo‐based TMDs over prolonged periods of laser exposure.^[^ ^115^ ^]^ Additionally, Peimyoo et al. successfully fabricated a high‐quality HfO_x_ layer by employing laser‐assisted oxidation on HfS_2_ flakes.^[^ ^121^ ^]^

Another advanced technique that enables selective oxidation is the application of local anodic oxidation (LAO) utilizing conductive AFM. In this method, liquid meniscus bridges are formed between the biased tip and the surface of the sample, driving the chemical reaction that leads to the oxidation of 2D TMD flakes, as depicted in Figure 6c.^[^ ^122^, ^123^, ^124^ ^]^ The size of the bridge can be adjusted by manipulating the applied bias between the AFM tip and the sample surface, thereby allowing precise control over the size of the oxidation features, as shown in Figure 6d, which displays an array of WO_x_ dots with varying sizes.^[^ ^109^, ^122^, ^125^ ^]^ It is worth noting that a short pretreatment of the TMD surface with oxygen plasma prior to the AFM‐assisted oxidation process allows for sub‐10 nm patterning of few‐layer TMDs.^[^ ^126^ ^]^

**Table** **1** summarizes different oxidation techniques we discussed in this section in terms of cost, throughput, precision, oxidation strength, and self‐limiting properties. These methods have demonstrated the ability to achieve precise nanoscale oxidation profiles compared to other techniques. However, these approaches require complex equipment and exhibit relatively low yields, rendering them unsuitable for practical applications. Consequently, there are ample opportunities to develop advanced oxidation techniques that can deliver high‐quality, precise, and selectively patterned oxide layers through simplified and cost‐effective processes.

**Table 1:** Comparison of oxidation techniques in terms of cost, throughput, precision, oxidation strength, and self‐limiting properties.

	Air exposure	O_2_ plasma	UV‐ozone	Annealing	AFM/laser
Cost	Low	High	Middle	Low	High
Throughput	Slow	Fast	Fast	Middle	Slow
Precision	Low	High	High	Low	High
Strength	Mild	Strong	Strong	Strong	Medium
Self‐limiting	Low	High	High	Low	Low

## 4 Applications

### 4.1 Charge Transfer Doping by Oxidation

**Figure 7:**
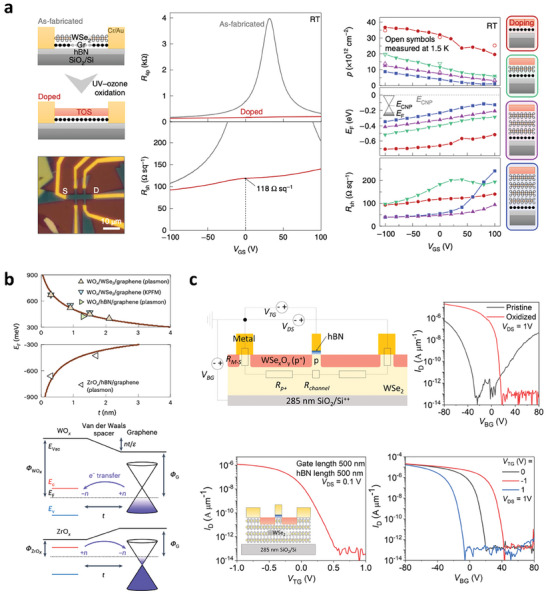
a) Charge transfer doping of graphene in WO_x_/graphene heterostructures. Reproduced with permission.^[^ ^19^ ^]^ Copyright 2021, Springer Nature. b) Tunable charge transfer doping of graphene using vdW interlayers: p‐ and n‐type doping using WO_x_ and ZrO_x_ charge‐transfer dopants, respectively.^[^ ^113^ ^]^ Copyright 2023, Springer Nature. c) Self‐aligned vdW top gate combined with local WO_x_ dopants near electrical contacts. Reproduced with permission.^[^ ^130^ ^]^ Copyright 2023, American Chemistry Society.

Oxidized TMDs span a wide range of work functions depending on the type of transition metal ions and their oxidation states.^[^ ^127^ ^]^ This attribute can be leveraged to dope 2D materials by placing oxidized TMDs with a high work function on the surface of 2D materials and creating a large work‐function mismatch to drive charge transfer. For example, Choi et al. demonstrated that graphene can be doped to ≈660 meV by oxidizing WSe_2_/graphene into WO_x_/graphene using self‐limiting oxidation techniques, in which a high‐work‐function WO_x_ hole dopes the underlying graphene (**Figure** **7**a).^[^ ^19^ ^]^ One advantage of this is that the pristine interfacial quality of WSe_2_/graphene is naturally transferred to WO_x_/graphene upon oxidation. As a result, the WO_x_/graphene interface is devoid of any interfacial absorbates or defects that could otherwise compensate for the work function mismatch and reduce the charge transfer.^[^ ^127^ ^]^ Kim et al. further showed that the same charge transfer technique can be applied to n‐type doped graphene by oxidizing ZrSe_2_/graphene to ZrO_x_/graphene (Figure 7b).^[^ ^113^ ^]^ In this structure, the smaller ZrO_x_ work function than that of undoped graphene results in the electron doping of graphene. The magnitude of the induced charge density can also be systematically tuned by inserting atomically inert layers of WSe_2_ to increase the distance between the oxidized TMDs and graphene. It was also shown that creating WO_x_‐encapsulated structures can further enhance the doping density in 2D materials by simultaneously initiating charge transfer at both the top and bottom interfaces.^[^ ^128^ ^]^ Furthermore, a unique electrostatic model was developed in the aforementioned studies to precisely predict the charge transfer at the WO_x_/graphene and ZrO_x_/graphene interfaces. This model only considers simple electrostatic boundary conditions between dissimilar materials, making it a powerful method for accurately predicting and designing charge transfer at vdW interfaces.

The same charge‐transfer doping technique has significant implications for the contact engineering of 2D materials. The electrostatic model described above can be used to quantitatively estimate charge transfer between the contact metal and 2D semiconductors. This information is essential for the precise analysis of the electrical contact characteristics of 2D materials. Borah et al. used oxidized WSe_2_ to improve the p‐type contact resistance of WSe_2_ FETs.^[^ ^129^ ^]^ In this study, the channel region of a multilayer WSe_2_ FETs was doped by oxidizing it into WO_x_. After doping, the electrical characteristics of the FETs were significantly enhanced, with an overall contact resistance of 642 Ω µm. Ngo et al. demonstrated a method for locally doping the contact region of WSe_2_ FETs using a deliberate combination of oxidation and a self‐aligned vdW top gate (Figure 7c). This approach enabled high‐performance 2D WSe_2_ p‐FETs exhibiting a high on/off ratio of 2.5 × 10^7^, SS of ≈98 mV dec^−1^, and high on‐state current of ≈100 µA.^[^ ^130^ ^]^ These examples highlight that oxidation techniques present a new route for engineering contact resistance and realizing advanced electronic device architectures incorporating 2D materials.^[^ ^131^ ^]^

### 4.2 Atomic Layer Etching

**Figure 8:**
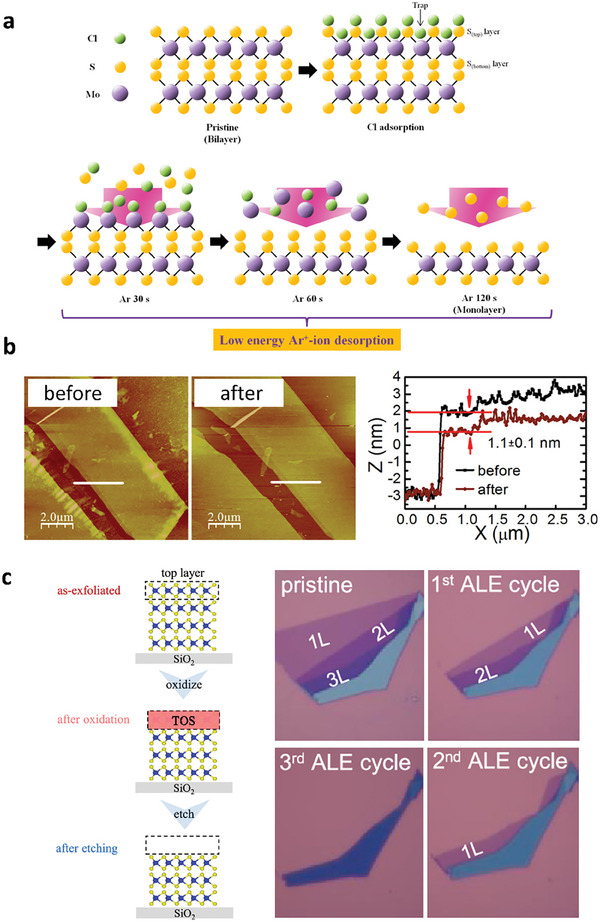
a) Illustration of the mechanism for the ALE process of MoS_2_ through Cl‐radical adsorption and Ar^+^‐ion desorption. Reproduced with permission.^[^ ^132^ ^]^ Copyright 2017, American Chemistry Society. b) AFM images and line profiles for MoS_2_ before and after ALE. Reproduced with permission.^[^ ^136^ ^]^ Copyright 2016, American Chemistry Society. c) Schematic for the ALE process that involves oxidation and the wet etching process. OM images of WSe_2_ with multiple cycles of ALE. Reproduced with permission.^[^ ^24^ ^]^ Copyright 2021, American Chemistry Society.

Atomic layer etching (ALE) of TMDs is a process used to selectively remove the layers of these materials with atomic precision. ALE is important for the fabrication of electronic devices and other nanoscale structures. The ALE process typically involves exposing the TMD surface to a reactive gas, such as fluorine or chlorine, which reacts with the top layer of the material to form a volatile product that can be removed from the surface.^[^ ^132^ ^]^ The reaction is stopped, and the surface is exposed to a second gas, such as argon or H_2_O, to remove any remaining volatile products and clean the surface, as illustrated in **Figure** **8**a.^[^ ^132^, ^133^, ^134^ ^]^ This cycle is repeated until the desired number of layers has been removed.

Alternatively, the use of remote or ICP with low kinetic energy reduces the chemical reaction between oxygen radicals and TMDs, thereby minimizing damage and preserving the structural integrity of the 2D material.^[^ ^24^, ^135^, ^136^ ^]^ Zhu et al. employed a two‐step etching process to achieve ALE of MoS_2_ through remote plasma treatment, resulting in the formation of a uniform MoO_3_ monolayer, followed by vacuum annealing at 500 °C. Figure 8b demonstrates that the etched step height measures ≈0.9‐1.1 nm, which closely matches the thickness of a monolayer of MoS_2_ (≈0.7 nm), indicating successful ALE.

One challenge in the ALE of TMDs is achieving selectivity, which refers to the ability to remove certain layers while leaving others intact. This can be achieved by tuning the gas chemistry and reaction conditions for selective reactions with certain layers of the material. UV‐ozone oxidation is a promising candidate for realizing ALE owing to the self‐limiting nature of oxidized TMDs. Several groups have used UV‐ozone oxidation to realize self‐limiting oxidation, as it is a mandatory process for ALE.^[^ ^24^, ^86^, ^90^ ^]^ Nipane et al. performed damage‐free atomic layer etching using room temperature UV‐ozone oxidation, which differs from previous studies that used long‐term ozone exposure without UV light.^[^ ^24^ ^]^ WO_x_ can be wet‐etched in KOH, providing excellent etching selectivity compared to its parent WSe_2_ flakes. This etching selectivity allows the ALE of the WSe_2_ layers by performing a series of oxidizing and etching steps in a layer‐by‐layer fashion, as shown in Figure 8c. ALE can also be employed to effectively remove surface polymer residues from 2D materials, with WSe_2_ serving as the sacrificial layer. The UV‐ozone treatment oxidizes WSe_2_, protecting the underlying layer while simultaneously eliminating surface polymer residues. The subsequent removal of the oxide layer through KOH treatment enables the fabrication of high‐mobility 2D FETs because of the clean surface. Overall, the ALE of TMDs is an important tool for fabricating and designing high‐performance nanoscale 2D devices with precise control over the number of layers.

### 4.3 Memory Devices for Neuromorphic Computing

**Figure 9:**
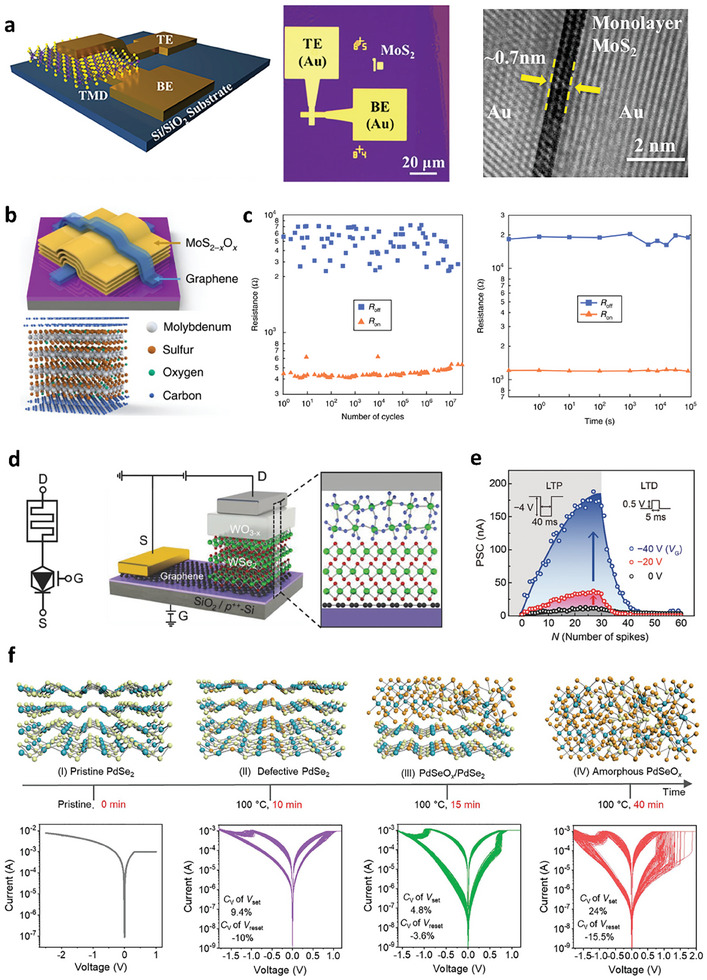
a) Schematic diagram, OM, and cross‐sectional TEM images for a MoS_2_ atomic memristor. Reproduced with permission.^[^ ^137^ ^]^ Copyright 2018, American Chemistry Society. b,c) Memristor with a graphene/MoS_2‐x_O_x_/graphene vertical heterostructure and its endurance and retention characteristics. Reproduced with permission.^[^ ^143^ ^]^ Copyright 2018, Springer Nature. d,e) Synaptic barristor fabricated using oxidized TMDs, showing efficient gate‐tunable synaptic behaviors. Reproduced with permission.^[^ ^25^ ^]^ Copyright 2018, Wiley‐VCH. f) Controlled resistive switching behaviors for a PdSeO_x_/PdSe_2_ heterostructure under different ozone treatment conditions. Reproduced under the terms of the CC‐BY 4.0 license.^[^ ^145^ ^]^ Copyright 2022, The Authors, Published by Wiley‐VCH.

With the increasing trend of data volumes, the von Neumann architecture can no longer guarantee the necessary processing speed and energy efficiency. Thus, a brain‐inspired, non‐von Neumann device structure has been proposed as a potential remedy for the von Neumann bottleneck. Generally, memristors are fabricated with active channels sandwiched between the bottom and top electrodes (BE and TE), as shown in **Figure** **9**a.^[^ ^137^ ^]^ To obtain memristors with low power consumption and high computing speed, the active layer must be thinned, requiring an ultrathin active layer. In this sense, 2D materials can be employed as the active components of memristors, realizing reduced power consumption and improved performance with small dimensions.^[^ ^138^, ^139^, ^140^ ^]^ Furthermore, the thinness of 2D materials allows for better control of charge transport within the device, resulting in faster switching speeds, higher storage densities, and gate‐tunabilities.^[^ ^141^, ^142^ ^]^

Recently, several studies have utilized standard oxidation procedures such as oxygen plasma or UV‐ozone treatment to prepare oxidized TMDs as memristive layers for 2D memristors. Wang et al. demonstrated robust memristors composed of a graphene/MoS_2‐x_O_x_/graphene vertical heterostructure (Figure 9b) produced by ambient air oxidation of MoS_2_ at 160 °C. This structure exhibited excellent switching performance with a good endurance up to ≈10^7^ cycles and retention up to 10^5^ sec, as shown in Figure 9c.^[^ ^143^ ^]^ Furthermore, the thermal stability and flexibility of the oxide layer are responsible for the high thermal operating temperature up to 340 °C and robust operation within 1000 bending cycles. Similarly, Alam et al. successfully demonstrated the fabrication of wafer‐scalable single‐layer MoO_x_ memristors by the UV‐ozone oxidation of monolayer MoS_2_ and a lithography‐free fabrication process.^[^ ^88^ ^]^ The role of mild UV‐ozone treatment is comparable to that of high‐energy oxygen plasma treatment in achieving local defect‐free single‐layer MoO_x_ memristors. Moreover, the flexible body of the oxidized TMDs may allow the fabrication of flexible electronic devices with high mechanical endurance that cannot be achieved with brittle oxide‐based memristors. Notably, oxygen plasma treatment can transform hBN into oxidized boron, which can be used as a weight‐control layer in synaptic devices.^[^ ^144^ ^]^

Huh et al. fabricated another vertical structure device utilizing oxidized WO_x_ as the active layer, as depicted in Figure 9d.^[^ ^25^ ^]^ By varying the work function of graphene, they proposed synaptic barristors with graphene as the BE and Ag as the TE sandwiching oxidized WSe_2_, whose memristive behavior strongly depends on the gate voltage, as shown in Figure 9e. Furthermore, using a unique oxidation procedure, lateral junction devices consisting of WSe_2_‐WSe_(2‐x)_O_y_‐WO_3_ fabricated from pristine WSe_2_ flakes showed clear optically stimulated synaptic plasticity, which is difficult to obtain using conventional oxide‐based vertical memristors.^[^ ^102^ ^]^

The resistive switching mechanism of the aforementioned device structure is mainly due to the migration of vacancies. For memristors working with other mechanisms, such as filament formation, the variation in the switching voltage has a significant impact on the computing accuracy. To solve this problem, Li et al. proposed a vertical device structure with oxidized PdSe_2_, forming a PdSeO_x_/PdSe_2_ heterostructure that exhibited uniform switching behavior with low variation in the SET and RESET voltages (Figure 9f).^[^ ^145^ ^]^ In a similar vein, C. Xiong et al. proposed the use of elemental selenium atoms that form at the TiO_2_/TiSe_2_ interface of an oxidized TiSe_2_ layer.^[^ ^146^ ^]^ Upon mild annealing (T < 200 °C), these selenium atoms coalesce and form nanocrystals. Memristor devices can be fabricated by subsequently capping this structure with another TiSe_2_ layer, resulting in TiSe_2_/TiO_2_/TiSe_2_ structures with selenium nanocrystals embedded at the bottom TiO_2_/TiSe_2_ interface. It was shown that applying an electric field vertically across TiSe_2_/TiO_2_/TiSe_2_ selectively drives conductive filament formation only near these nanocrystals, rendering an extremely robust cycle‐to‐cycle consistency of the device switching. In conclusion, the utilization of oxidized TMDs as the channel material for memristors remedies several difficulties toward high‐performance non‐von Neumann devices.

### 4.4 Bandgap Engineering and Quantum Confinement Effects

**Figure 10:**
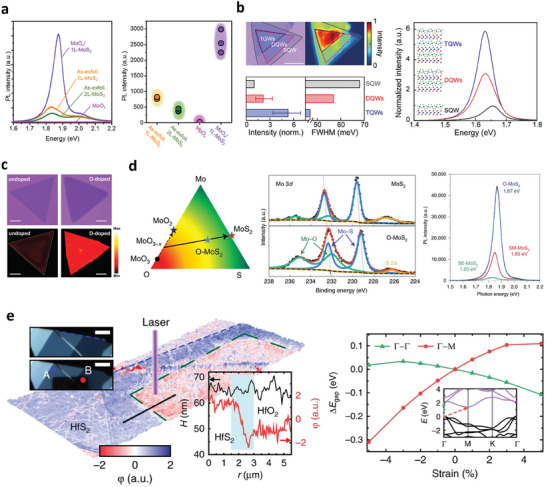
a) PL of the as‐exfoliated 1L‐MoS_2_, 2L‐MoS_2_, MoO_x_, and MoO_x_/MoS_2_. Integrated PL intensity of the as‐exfoliated 1L‐MoS_2_, 2L‐MoS_2_, MoO_x_, and MoO_x_/1L‐MoS_2_. Reproduced with permission.^[^ ^79^ ^]^ Copyright 2021, American Chemistry Society. b) Optical image (left) and spatially resolved PL map (right) of the step‐like stacked MQWs. PL spectra of the SQWs (black line), DQWs (red line), and TQWs (blue line). Histograms for the integrated PL intensity (left) and full width half maximum (FWHM) (right) for the SQWs (gray), DQWs (red), and TQWs (blue). Reproduced under the terms of the CC‐BY‐NC 4.0 license.^[^ ^147^ ^]^ Copyright 2021, The Authors, Published by American Association for the Advancement of Science. c) Optical images and the corresponding integrated PL intensity mappings of O‐doped and undoped WS_2_ monolayers. Scale bars in images are 20 µm. Reproduced with permission.^[^ ^152^ ^]^ Copyright 2021, Wiley‐VCH. d) Ternary phase diagram of MoS_2_, indicating the possible reaction routes for the CVD growth of MoS_2_. XPS data of Mo 3*d* for a typical CVD MoS_2_ film grown under the sulfur‐mild condition, and an O‐MoS_2_ film showing the presence of Mo–O bonds in O‐MoS_2_. Typical PL spectra of O‐MoS_2_, SM‐MoS_2_, and SE‐MoS_2_ flakes grown on SiO_2_/Si substrates acquired in an ambient environment, showing the PL enhancement of O‐MoS_2_. Reproduced with permission.^[^ ^153^ ^]^ Copyright 2022, Springer Nature. e) AFM topography of HfS_2_ flake after laser exposure. The insets are optical images before and after laser‐assisted oxidation. Scale bars are 5 µm. Compressive strain was induced in the center of a semiconducting HfS_2_ channel by controlled photo‐oxidation. The compression induced tensile strain away from the HfS_2_/HfO_2_ interface, resulting in the spatial modulation of the bandgap. The change in bandgap was represented as a function of strain in the two directions, with respect to the unstrained bandgap (relaxed lattice constant *a*_0_  =  3.625 Å). Inset: calculated band structure of 1T‐HfS_2._ Reproduced under the terms of the CC‐BY 4.0 license.^[^ ^26^ ^]^ Copyright 2018, The Authors, Published by Springer Nature.

The oxidation of 2D TMDs contributes to the strong bandgap modulation of this group of materials. The controllable oxidation of multilayer TMDs to obtain oxide/1L TMDs induces an indirect‐to‐direct bandgap transition due to the quantum confinement effect.^[^ ^79^, ^90^, ^147^, ^148^ ^]^ Kang et al. proposed an optimal oxygen plasma condition to introduce monolayer MoO_x_ on top of monolayer MoS_2_ from bilayer MoS_2_, which contributes to the enhancement of PL intensity compared to the exfoliated monolayer MoS_2_, as shown in **Figure** **10**a.^[^ ^79^ ^]^ This latter effect arises from the suppression of trion peaks, which originate from S vacancies or the substrates, attributed to the de‐doping effect from the top oxide layer. In WSe_2_, the transition from an indirect to a direct bandgap was also realized in oxide/1L WSe_2_ heterostructures by oxidizing multilayer WSe_2_ flakes.^[^ ^90^, ^147^ ^]^ Furthermore, a monolithic combination of several oxide/1L TMDs stacks led to atomic‐layer‐confined multiple quantum‐well band structures, providing ultra‐high PL intensity compared to that of intrinsic TMDs with similar thickness (Figure 10b).^[^ ^79^, ^147^ ^]^

However, the PL quenching of monolayer MoS_2_ was observed via oxygen plasma treatment owing to the substitution of S atoms on MoS_2_ crystals by O atoms, leading to a direct‐to‐indirect bandgap transition.^[^ ^149^ ^]^ Thus, the engineering of the bandgap of 2D TMDs by oxidation must be carefully conducted without a direct high‐energy bombardment to 2D TMD crystals. Moreover, mild oxidation treatment healed chalcogenide‐based vacancies by chemically adsorbed oxygen atoms; these deactivate the defect‐induced gap states by donor defects such as chalcogenide‐based vacancies. Nan et al. adopted mild oxygen plasma treatment (13.56 MHz, 5 W, 5 Pa) to heal donor defects, resulting in improvement in neutral exciton intensity.^[^ ^150^ ^]^ Lu et al. improved the photo responsivity of CVD‐grown WSe_2_ FETs by healing Se vacancies utilizing mild laser‐assist oxidation treatment in air.^[^ ^151^ ^]^ Similarly, by employing thermal annealing in oxygen at low pressure (0.5 Pa), Liu et al. healed Te vacancies by chemically intercalated oxygen atoms to obtain the ideal bandgap of trilayer MoTe_2_ (≈1 eV) instead of a defective bandgap (≈0.72 eV).^[^ ^106^ ^]^

In addition to defect engineering by top‐down oxidation techniques, oxygen is provided to fill the chalcogenide vacancies during the synthesis of TMDs by CVD growth. Cui et al. introduced oxygen atoms during the CVD growth of WS_2_ by adding Fe_2_O_3_ powder in the quartz tube; the O‐doped WS_2_ demonstrated suppression of defective PL‐bound excitons corresponding to defects measured at 87 K. Therefore, fewer defect‐induced gap‐states were observed in O‐doped WS_2_ compared to in WS_2_ synthesized in the absence of oxygen, as depicted in Figure 10c.^[^ ^152^ ^]^ Similarly, instead of using a solid‐state oxygen source, Shen et al. introduced oxygen gas with Ar to realize O‐MoS_2_ with relatively fewer defect‐induced gap states, as demonstrated by the improvement in PL neutral exciton intensity and electrical performance (Figure 10d).^[^ ^153^ ^]^

Recently, in addition to bandgap engineering induced by the oxidation of 2D TMDs based on the quantum confinement phenomenon, the surface formation of HfO_2_ by the oxidation of HfS_2_ flakes induced an average compression strain of 2.7% at the interface of the bottom semiconducting HfS_2_ channel with its oxide, as shown in Figure 10e.^[^ ^26^ ^]^ The introduction of compression strain on the HfS_2_ lattice resulted in an enlargement of the direct bandgap and an opposite trend to that of the indirect bandgap. These findings suggest that oxidation techniques are an effective method for controlling the bandgap of TMDs.

### 4.5 Surface Functionalization for the ALD Process

**Figure 11:**
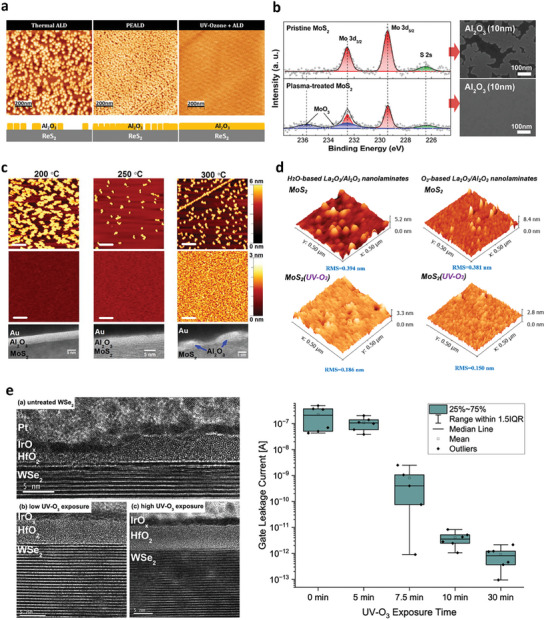
a) Ex situ AFM images of the following 80 cycles of ALD of Al_2_O_3_ with and without UV‐ozone treatment. Reproduced under the terms of the CC‐BY 4.0 license.^[^ ^157^ ^]^ Copyright 2019, The Authors, Published by MDPI Open Access Journal. b) XPS spectra of the Mo 3d and S 2s core levels measured from the pristine and plasma‐treated MoS_2_ surface and the corresponding SEM images of 10 nm ALD‐grown Al_2_O_3_ on top of the MoS_2_ flakes. Reproduced with permission.^[^ ^161^ ^]^ Copyright 2013, American Chemistry Society. c) AFM images of Al_2_O_3_ deposited by 30 ALD cycles at 200, 250, and 300 °C on exfoliated bulk MoS_2_ and oxygen functionalized MoS_2_ (O‐SMoS). HRTEM images of the corresponding Al_2_O_3_ film deposited on oxygen functionalized MoS2 (O‐SMoS). Scale bars in AFM images are 200 nm, and those in HRTEM images are 5 nm. Reproduced with permission.^[^ ^162^ ^]^ Copyright 2014, AIP Publishing. d) AFM results of sub‐5 nm La_2_O_3_/Al_2_O_3_ nanolaminates on MoS_2_ with or without UV‐O_3_ treatment. Reproduced under the terms of the CC‐BY 4.0 license.^[^ ^163^ ^]^ Copyright 2022, The Authors, Published by MDPI Open Access Journal. e) TEM images and leakage current of top‐gate WSe_2_ FETs with various UV‐ozone treatment times prior to ALD of the gate dielectric. Reproduced with permission.^[^ ^164^ ^]^ Copyright 2023, AIP Publishing.

High‐performance 2D FETs incorporating dual‐gated structures have emerged as promising candidates for computations beyond Moore's law. However, a significant challenge in realizing this potential lies in achieving a uniform oxide layer on 2D TMD flakes, which serve as high‐k dielectrics, through ALD processes. This challenge stems from the dangling bond‐free nature of the 2D TMD surface, which hinders covalent nucleation of the precursor on its surface.^[^ ^154^ ^]^ While a uniform thin film can be achieved through physical adsorption, a low‐temperature ALD process is necessary to prevent the release of physisorbed molecules, thereby avoiding poor density and excessive incorporation of impurities from the precursor.^[^ ^155^, ^156^ ^]^ Consequently, without surface functionalization, high‐k oxide films like HfO_2_ and Al_2_O_3_ formed on 2D TMDs via ALD exhibit non‐conformal characteristics, featuring several pinholes after multiple cycles of the ALD process (**Figure** **11**a).^[^ ^157^, ^158^, ^159^ ^]^ Therefore, oxidation is commonly employed as a surface functionalization strategy, inducing numerous  ‐OH species on the surface of TMD flakes to achieve uniform and conformal high‐k oxide layers.^[^ ^155^, ^156^ ^]^

Oxygen plasma has been employed to form hydrophilic MoO_x_ layers containing multiple ‐OH groups, facilitating improved coverage of high‐k oxides on the surface of Mo‐based TMDs (Figure 11b).^[^ ^160^, ^161^ ^]^ Nevertheless, when utilizing oxygen plasma to create thick MoO_x_ layers on 2D TMDs, the intrinsic electrical performance of the underlying ultrathin TMD layers may be affected by significant ion bombardment. Therefore, a UV‐ozone treatment was utilized to create surface dangling bonds without adversely affecting the integrity of the 2D TMD layer. Azcalt et al. investigated the effects of UV‐ozone treatment on Al_2_O_3_ grown via ALD on MoS_2_ (Figure 11c).^[^ ^162^ ^]^ The appearance of Mo‐S‐O, as revealed by in situ XPS, indicates that the functional groups formed after UV‐ozone treatment serve as nucleation sites for ALD‐grown Al_2_O_3_, resulting in uniform growth on the surface of the MoS_2_ flakes. Moreover, the absence of a Mo^6+^ peak in the XPS spectra suggests preservation of the intrinsic properties of the underlying MoS_2_ flakes. However, employing this technique at high temperatures exceeding 300 °C leads to the decomposition of oxygen in S‐O species generated during UV‐ozone treatment, which is unfavorable for high‐quality ALD processes. In Figure 11d, Fan et al. utilized UV‐ozone treatment prior to ALD at 260 °C for the La_2_O_3_/Al_2_O_3_ film on the surface of MoS_2_ flakes, resulting in formation of a uniform thin‐film oxide without pinholes and a low root mean square value of ≈0.15 nm, regardless of the oxidant used.^[^ ^163^ ^]^

Similar to MoS_2_, the exposure of MoSe_2_ and WSe_2_ to UV‐ozone leads to the formation of MoO_x_ and WO_x_, respectively.^[^ ^120^ ^]^ Following UV‐ozone treatment, a uniform ALD‐grown HfO_2_ thin film was successfully deposited on the surface of the oxidized MoSe_2_ flakes, reducing the oxidation state from Mo^6+^ to Mo^5+^, which is indicative of the self‐cleaning reaction of the MoO_x_ layer. In contrast, nonuniform HfO_2_ islands were formed on the oxidized WSe_2_, with WO_x_ species persisting on the surface of the flakes. Furthermore, Sales et al. proposed that a uniform thin HfO_2_ layer, ≈5 nm thick, could be achieved through thermal ALD with an adequate UV‐ozone treatment duration. This is in contrast to the inhomogeneous dielectric layer obtained using the same ALD process without surface functionalization via UV‐ozone treatment (Figure 11e).^[^ ^164^ ^]^ Consequently, the leakage current observed in top‐gate FETs treated with UV‐ozone was comparatively lower than that in those without such treatment.

Recently, gate‐all‐around (GAA) FETs based on 2D TMDs have garnered significant attention in both industry and academia.^[^ ^165^, ^166^, ^167^ ^]^ In order to ensure successful integration of 2D TMDs into future CMOS applications, including GAA FETS, it is critical to achieve compatibility of 2D TMDs with key CMOS technologies, particularly ALD processes. The surface functionalization of 2D TMDs by forming 2D TMOs on the surface uniquely provides a promising route for nucleation and growth directly on 2D materials during ALD processes. In particular, surface functionalization of 2D materials utilizes non‐directional gas‐phase methods, such as UV‐ozone treatment, remote plasma treatment, and oxygen annealing. This latter aspect further enhances the practicality of adopting these TMD oxidation processes in large‐scale manufacturing and in existing industrial practices. Thus, oxidized 2D TMDs may serve as a key enabler for building industrially compatible advanced 2D TMDs‐based GAA FETs for upcoming technology nodes.

Consequently, surface functionalization via oxidation has been systematically explored for MoS_2_. However, the application of high‐k oxides obtained by ALD on the surfaces of other TMDs using surface functionalization remains limited, necessitating further research.

### 4.6 Buffer Layer for the Contact Interface and Growth of High‐k Dielectrics

**Figure 12:**
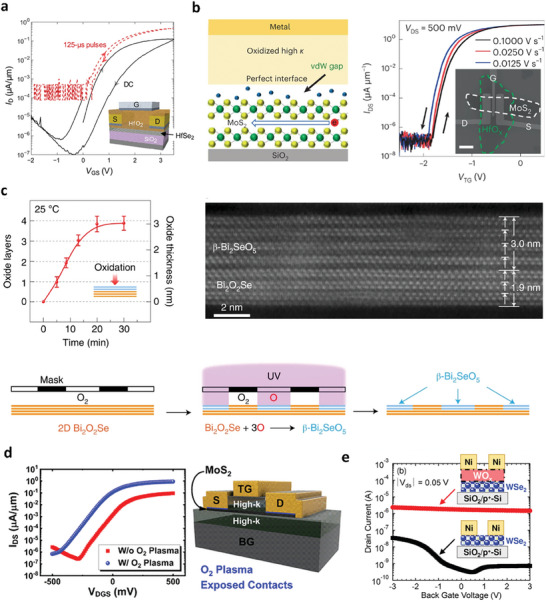
a) Top‐gated HfSe_2_ transistor with native HfO_2_ dielectric. Reproduced under the terms of the CC‐BY‐NC 4.0 license.^[^ ^27^ ^]^ Copyright 2017, The Authors, American Association for the Advancement of Science. b) Cross‐sectional representation of the gate interface states in MoS_2_ VGG transistors. Transfer curves of the VGG transistors with different gate‐voltage sweeping speeds. Reproduced with permission.^[^ ^93^ ^]^ Copyright 2022, Springer Nature. c) Layer‐by‐layer oxidation of 2D Bi_2_O_2_Se under vacuum‐UV (185 nm) exposure at room temperature. Cross‐sectional STEM‐HAADF image of a uniform ultrathin lattice‐matched single‐crystalline 2D Bi_2_O_2_Se/β‐Bi_2_SeO_5_ heterostructure. Area‐selective oxidation with UV and photolithography mask free of polymers. Reproduced with permission.^[^ ^171^ ^]^ Copyright 2022, Springer Nature. d) Graphic cross section of a dual‐gate (DG) MoS_2_ FET with a HfO_2_/Al_2_O_3_/MoS_2_/Al_2_O_3_ gate stack. Comparison of transfer curves of DG MoS_2_ FETs demonstrating a similar, near‐ideal SS of ∼60 mV/dec with over an order of magnitude increase in the ON current for devices with O_2_ plasma exposure at the contacts. Reproduced with permission.^[^ ^77^ ^]^ Copyright 2019, American Chemistry Society. e) *I*_d_ –*V*_g_ characteristics of WSe_2_ FETs with and without a buffered‐WO_x_ layer. Reproduced under the terms of the CC‐BY 4.0 license.^[^ ^175^ ^]^ Copyright 2023, The Authors, Published by IEEE.

Short‐channel effect is a significant challenge that impedes the downscaling of integrated electronics incorporating 2D materials. Integrating high‐k dielectrics into electronic circuits can mitigate this effect by simultaneously scaling down the effective oxide thickness, which will enable higher speed and low power consumption.^[^ ^168^ ^]^ TMOs are ideal high‐k dielectric candidates, but integrating TMOs into 2D semiconductors without perturbing their intrinsic quality (mobility) is challenging because of the dangling bond‐free interface of 2D TMDs.^[^ ^120^, ^155^, ^156^ ^]^ Either direct deposition or ALD of TMO directly introduces damage to the lattice sites of 2D semiconductors, ultimately limiting channel mobility. In this regard, using oxidation to transform HfSe_2_ into HfO_x_ has been proposed as an effective approach to integrate high‐k dielectrics into device platforms without compromising the channel mobility, which is essential for high‐performance logic applications.^[^ ^27^ ^]^ Integration of a native high‐k dielectric layer onto HfSe_2_ or ZrSe_2_ by oxidation allows the fabrication of high‐performance top‐gate FETs low‐voltage operation and a sufficiently large on/off ratio of 10^6^, as depicted in **Figure** **12**a. Kang et al. also showed that a high‐quality HfO_2_/HfSe_2_ gate produced by oxidizing HfSe_2_ enables an impact ionization FETs with ultra‐steep subthreshold swing of 3.43 mV dec^−1^ at room temperature.^[^ ^169^ ^]^ In a similar vein, Lou et al. and Jing et al. utilized oxidation of a HfS_2_ flake to form a high‐k HfO_x_ layer on a MoS_2_ flake to fabricate top‐gate MoS_2_ FETs.^[^ ^93^, ^170^ ^]^ As a result, a widened vdW gap was observed at the interface of HfO_x_ and MoS_2_, which contributed to the ideal interface between the 2D semiconductor and insulator. This resulted in a negligible hysteresis of 10 mV and close to thermionic limit SS of 53.1 mV dec^−1^, as shown in Figure 12b.^[^ ^93^ ^]^

Recently, Zhang et al. demonstrated the use of UV‐ozone treatments at room temperature to synthesize high‐k dielectric β‐Bi_2_SeO_5_, which can achieve equivalent‐oxide‐thickness below 0.5 nm with low leakage current, satisfying the IRDS roadmap.^[^ ^171^, ^172^ ^]^ This study also demonstrated local oxidation using a hard mask and UV‐assisted layer‐by‐layer oxidation process, which is potentially compatible with industrial‐level grey‐scale lithography. Thus, a desirable high‐k oxide pattern can be obtained for large‐scale CMOS applications (Figure 12c). The same research group employed this method to nucleate the gate stack for high‐performance novel FinFETs, thereby presenting opportunities to extend Moore's law.^[^ ^173^, ^174^ ^]^

In addition, the utilization of oxidized TMDs as buffer layers can improve the contact interface between 2D semiconductors and metals. Bolshakov et al. performed an oxygen plasma treatment to form MoO_x_ at the contact area prior to metal evaporation, which resulted in Ohmic‐like behavior in the device (Figure 12d).^[^ ^77^ ^]^ Moreover, unipolar p‐type behavior was obtained by forming the WO_x_ layer near the contact region before metal contact deposition for WSe_2_ FETs, as shown in Figure 12e. Using this approach, p‐type FETs with high on/off ratios can be realized without requiring sophisticated doping patterns.^[^ ^175^, ^176^ ^]^

### 4.7 Controlling Light‐Matter Interactions in 2D Materials

**Figure 13:**
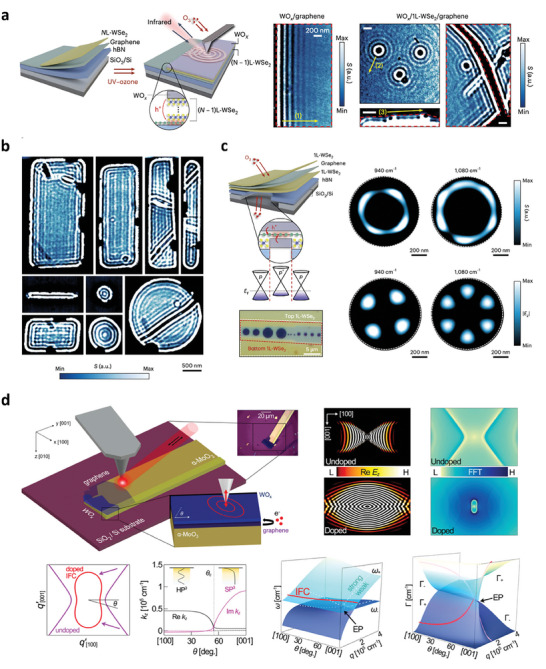
a) Nano‐infrared imaging of charge‐transfer plasmon polaritons in WO_x_/graphene heterostructures. “Programmable” nano‐patterned WO_x_/graphene heterostructures exhibit b) confined plasmonic nano‐cavity modes and c) whispering‐gallery modes. Images in a)‐c) reproduced with permission.^[^ ^113^ ^]^ Copyright 2023, Springer Nature. d) Electronically‐induced topological transition and exceptional‐point singularities in coupled plasmon‐phonon modes of WO_x_/graphene/a‐MoO_3_ heterostructures. Reproduced under the terms of the CC‐BY 4.0 license.^[^ ^177^ ^]^ Copyright 2022, The Authors, Published by Springer Nature.

Light‐matter interactions in 2D materials can be drastically engineered by taking leverage of the charge transfer at the oxidized TMD/graphene interface. For example, oxidation‐activated charge transfer (OCT) in WO_x_/graphene produces charge‐transfer plasmon polaritons that propagates a long‐range distance along the WO_x_/graphene interface (**Figure** **13**a,b).^[^ ^113^ ^]^ Kim et al. harnessed this OCT to program low‐loss plasmonic nanocavities at target locations of continuous monolayer graphene with performance metrics limited by their intrinsic properties. These plasmonic cavities exhibit laterally abrupt doping profiles with previously unattainable single‐digit nanoscale precision. Furthermore, technologically appealing but elusive plasmonic whispering‐gallery cavities were demonstrated, for the first time, based on graphene using suspended structures of WO_x_/graphene/WO_x_ (Figure 13c). These modes arise from edge plasmons looping around the cavity circumference and require laterally sharp cavity boundary, demonstrating the superiority of OCT methods in producing laterally sharp plasmonic nanostructures.

The OCT‐induced plasmon polaritons can also be leveraged to induce exotic optical phenomena in proximal vdW layers. F. Ruta et al. demonstrated electronically‐induced topological transition in the isofrequency surface (IFS) of a‐MoO_3_ phonon polaritons by oxidizing WSe_2_/graphene/a‐MoO_3_ into WO_x_/graphene/a‐MoO_3_ heterostructures, as shown in Figure 13d.^[^ ^177^ ^]^ Before oxidation, graphene remains nominally undoped, and a‐MoO_3_ phonon polaritons displays hyperbolic IFS. Upon oxidation, isotropic graphene plasmons emerge and strongly couple with a‐MoO_3_ phonon polaritons to induce a topological transition, forming a hybrid plasmon‐phonon mode with a closed, elliptical IFS. More interestingly, exceptional point (EP) singularities were shown to exist in the same structures as a result of the propagation‐direction‐dependent coupling strength of graphene plasmons with a‐MoO_3_ phonon polaritons. EPs in optical systems are of great interest since they exhibit many exotic yet useful optical phenomena,^[^ ^178^ ^]^ including parametrically amplified sensitivity for unparalleled sensing and imaging capabilities.^[^ ^179^, ^180^ ^]^ In WO_x_/graphene/a‐MoO_3_ structures, coupled plasmon‐phonon modes can be precisely tuned near EP by tuning the propagation direction of the hybrid plasmon‐phonon modes. Thus, charge‐transfer plasmons in WO_x_/graphene sets a strong foundation for implementing a next generation of sensing technologies and exploring EP physics in non‐Hermitian optical systems based on 2D heterostructures.

### 4.8 Photonic Applications

Exploiting the physical coupling between 2D materials and their oxidized forms with photonic structures can enable new photonic applications. Kim et al. fabricated multiple quantum wells (MQWs) using type‐I band‐aligned WO_x_/WSe_2_ heterobilayers and reported a superlinear increase in PL while increasing the number of 2D QWs in vertical heterostructures.^[^ ^147^ ^]^ These monolithic MQWs have great potential for applications in high‐performance light‐emitting devices such as light‐emitting diodes (LEDs), lasers, and single‐photon emitters. Using oxidized TMDs, effective optoelectronic devices can be developed through band engineering, native high‐k dielectric, and doping engineering mentioned above. Importantly, these devices can be easily integrated and coupled into photonic structures, opening new opportunities for new forms of photonic integrated circuits (PICs).^[^ ^11^, ^12^, ^181^ ^]^ Examples include optical waveguides, optical cavities, distributed Bragg reflectors (DBR), photonic crystals, and metasurfaces that can improve the performance of optical devices and enable new functions through light confinement and guiding.

**Figure 14:**
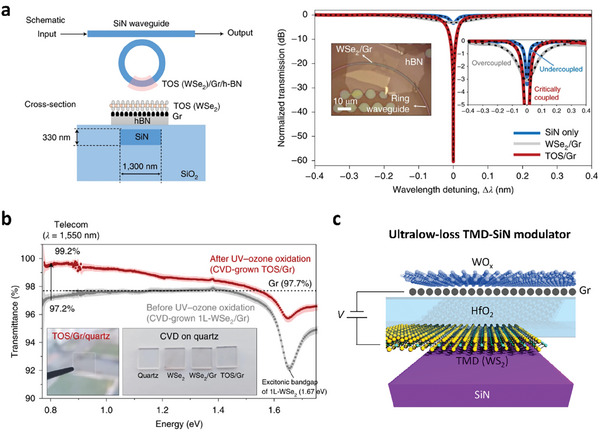
a) Microring resonator integrating WO_x_/graphene/hBN into SiN waveguide. Resonator transmission spectra was tuned to critical coupling condition after UV–ozone oxidation of WSe_2_, showing the reduction of insertion loss of graphene by WO_x_ doping. Reproduced with permission.^[^ ^19^ ^]^ Copyright 2021, Springer Nature. b) Transmittance of intrinsic graphene (97.7%) increase to 99.2% using a WO_x_ layer at telecommunication wavelength (1550 nm). Reproduced with permission.^[^ ^19^ ^]^ Copyright 2021, Springer Nature. c) Schematic diagram of ultralow‐loss phase tuning of monolayer WS_2_ using the WS_2_–HfO_2_–WO_x_/graphene capacitor structures. Monolayer WS_2_ can be electrostatically doped by applying a bias voltage between two electrodes (TMD and WO_x_/graphene) across the dielectric HfO_2_, which changes the refractive index while minimizing absorption.

Choi et al. demonstrated the integration of oxidized WSe_2_/graphene/hBN structures on planarized SiN waveguides with microring resonator cavities (**Figure** **14**a).^[^ ^19^ ^]^ Before the integration of WO_x_/graphene, the ring transmission spectrum of the bare low‐loss cavity displayed weak coupling of microring resonators to the straight waveguide (uncoupled region). After incorporating WSe_2_/graphene/hBN on a planarized SiN substrate, a high insertion loss was induced in undoped graphene, leading to a region with overcoupled cavity coupling when the resonator linewidth is broadened. After UV‐ozone oxidation, the interband absorption of graphene was strongly suppressed due to WO_x_ doping, resulting in lower insertion loss of 0.012 dB µm^−1^. In particular, a critically coupled resonance transmission response was demonstrated, suggesting that WO_x_‐doped graphene can perform as a powerful alternative to existing transparent conductive materials such as ITO. These latter materials demonstrate insertion loss of 1.6 dB µm^−1^.^[^ ^182^ ^]^ Moreover, from the transmission spectrum in Figure 14b, the near‐infrared transmittance of graphene was noticeably enhanced with WO_x_ doping from 97.7% to 99.2% at the technologically relevant telecommunication wavelength (λ = 1550 nm). While conventional transparent conductors typically suffer from a tradeoff between transmittance and sheet resistance, WO_x_/graphene and WO_x_/3L‐WSe_2_/graphene offer extremely high transmittance while maintaining very low sheet resistance. Such a low absorption and sheet resistance establish WO_x_/graphene as an attractive electrode material platform for integrated photonics. Datta et al. demonstrated that the properties of monolayer TMDs can be modified by applying electrical gating using ionic liquids [P14^+^] [FAP^−^] to control the optical phase by adjusting the effective mode index (*ñ* = *n* + i*k*, where the *n* is a real part and *k* is an imaginary part).^[^ ^182^ ^]^ At NIR wavelengths, gating induced a strong electrorefraction response (*Δn*) and a small electroabsorption response (*Δk*) of the monolayer TMDs, which showed low insertion loss despite the large phase change of the propagating light. When the ionic liquid is replaced with a TMD‐HfO_2_‐ITO capacitor, insertion loss is increased by ≈130 times as a result of absorption of ITO. Interestingly, replacing ITO gate electrode with WO_x_/graphene could enable a solid‐state TMD phase modulator with minimized insertion loss (Figure 14c). Therefore, oxidized TMDs has a high potential to drive the advancement of future photonic applications, including high‐speed optical modulation, frequency shifting, nonlinear optics, sensing, and laser devices.

In **Table** **2**, we summarize the applications of oxidized TMDs achieved through various oxidation techniques. Unlike air exposure and AFM/laser‐based oxidation, UV‐ozone and oxygen plasma treatments have been predominantly used due to their reproducibility and reliable processability. Their compatibility with CMOS technology also makes them suitable for wafer‐scale TMD applications. Further explorations of oxidation by annealing and AFM/laser oxidation in a broader application base, including surface functionalization and atomic layer etching, are promising next steps in expanding the potential of oxidized TMDs.

**Table 2:** Applications of oxidized TMDs and the corresponding oxidation techniques employed.

	Charge transfer doping	Atomic layer etching	Memory	Bandgap engineering	Surface functionaliz‐ation	Buffer layer	Light matter interaction
Air exposure			O			O	
O_2_ plasma	O	O	O	O	O	O	
UV‐ozone	O	O	O		O	O	O
Annealing			O	O			
AFM/laser				O			

## 5 Challenge and Outlook

As described in the preceding sections, oxidized TMDs is a rapidly growing field together with the recent development of novel surface engineering technologies that enable oxidation of TMDs down to their single monolayer limit. Looking into the future, we envision that oxidized TMDs will drive rapid advancements across various application fronts yet with several key challenges that must be overcome for their practical use. One relevant and important application front is the use of oxidized TMDs to tackle the long‐standing issue of making high quality electrical contacts to TMDs. This issue is a major roadblock that hinders the integration of 2D materials for future electronic devices, and oxidized TMD layers can serve as an essential vehicle to significantly improve contact resistance in 2D materials‐based devices.^[^ ^175^, ^176^ ^]^ For example, oxidized TMDs can be inserted as buffer layers prior to metal contact deposition to preserve the crystalline integrity of 2D materials at the metal‐semiconductor (MS) interface. This suppresses the generation of defect‐induced gap states.^[^ ^183^ ^]^ The same oxidized layer can also serve as additional dielectric layers at the contact to prevent orbital overlap between the contact metal and TMD channel, thereby circumventing metal‐induced gap states.^[^ ^184^ ^]^ Another advantageous characteristic of oxidized TMD layers is their ability to avoid direct introduction of defects or strain to the 2D materials, particularly underneath the contact region. These imperfections could otherwise lead to FLP that can drastically degrade the contact quality.^[^ ^184^ ^]^ However, unexpectedly formed oxidized layers on TMDs during fabrication processes can themselves be detrimental to the MS interface. For example, unintentionally formed oxide layers have shown to be the main source of issues in 2D‐based FETs, ranging from Schottky barrier height inhomogeneities to FLP.^[^ ^185^, ^186^, ^187^ ^]^ Thus, we anticipate that substantial efforts should be devoted to developing new technologies that can controllably trigger or inhibit the formation of oxide layers on TMDs on demand during the entire course of device fabrication steps.

Next important subject is achieving a precise control over patterned oxidation profiles on TMD surfaces to obtain controllable doping profiles in the underlying TMD layers. Un‐patterned oxidation of TMDs typically results in a degenerate p‐type behavior and impedes gate tunability, particularly in FETs, making it unfavorable for logic circuit applications.^[^ ^108^, ^129^, ^188^ ^]^ Early efforts have focused on laser‐assisted or local anodic oxidation techniques to create locally oxidized structures.^[^ ^109^, ^110^ ^]^ But these methods require complicated equipment setup and have relatively low yield and thus, are not suitable for practical applications. An alternative economic approach is covering the untreated area of TMDs with a mask layer (e.g., a photoresist or hBN) followed by a subsequent oxidation to create spatially confined oxidation profiles.^[^ ^113^, ^129^, ^188^, ^189^, ^190^, ^191^, ^192^, ^193^ ^]^ High‐quality p‐n or p‐n‐p lateral junctions have been shown using these latter methods. But using transferred hBN to realize desired oxidation profiles poses challenge for achieving ultra‐narrow doping profile due to the inherent misalignment errors during the transfer process.^[^ ^129^, ^191^ ^]^ Likewise, patterning oxidation profile using lithography processes may lead to the degradation of oxide layers due to the inherent exposure to solvents and ambient environment.^[^ ^89^, ^128^ ^]^ Thus, more work is needed to develop facile methods that enable spatial control of oxidation profile while effectively passivating the resultant oxide layers at the same time to prevent degradation.

Another sough‐after ability is controlling the oxidation depth of TMDs. Oxidized layers on TMDs can serve as electrolytes, functionalized surface layers for ALD processes, or high‐k dielectrics.^[^ ^66^, ^121^, ^156^ ^]^ But limited control over the oxidation depth is currently one major roadblock that impedes integrating oxidized TMDs into these applications. As elucidated in the preceding section on oxidation techniques, the oxidation depth of group VI TMDs can be effectively modulated layer‐by‐layer using oxygen plasma or UV‐ozone treatments combined with appropriately adjusted temperatures. However, advanced technologies that allow for precise oxidation depth control over a broad class of TMDs still remains to be developed. In particular, achieving layer‐by‐layer oxidation of group VI TMDs is challenging using current technologies as the oxidized layers on these TMDs do not prevent ozone from reaching the underlying pristine TMD layers.^[^ ^81^, ^93^, ^104^ ^]^ Interestingly, Luo et al. conducted the oxidation of an HfS_2_ flake stacked on MoS_2_ FETs, revealing undamaged MoS_2_ even after complete oxidation of HfS_2_ due to widened vdW gap from oxygen accumulation at the HfO_x_/MoS_2_ heterointerface.^[^ ^93^ ^]^ Thus, the use of hetero‐bilayers of TMD structures could be a potential future direction for tackling the oxidation depth control problem, particularly in VI TMDs.

Last but not the least important issue is the stability of TMO layers obtained by oxidizing TMDs and their compatibility with back‐end‐of‐line processes, which is still under much debate in the field. First, the thermal stability of oxidized TMDs is particularly important as the amorphous‐to‐(poly)crystalline phase transitions can adversely affect their dielectric properties and potentially compromise electronic device performance.^[^ ^194^ ^]^ Studies have shown that amorphous MoO_x_ formed on the surface of oxidized MoS_2_ tends to crystallize into α‐MoO_3_ at ≈450 °C.^[^ ^61^, ^98^, ^99^ ^]^ Likewise, TMOs formed on oxidized group IVB TMDs, such as HfO_x_ or ZrO_x_, crystallize at a similar temperature range of ≈450 °C.^[^ ^195^, ^196^, ^197^ ^]^ In contrast, WO_x_ on the surface of oxidized WSe_2_ is relatively stable, only crystallizing into WO_3_ above 600 °C,^[^ ^198^ ^]^ suggesting that thermal stability of oxidized TMDs varies from member to member. Thus, accumulating an exhaustive literature of thermal stability that encompass broader members of TMDs is required to better understand the precise nature of their thermal stability. A related issue is the chemical and doping stability. For example, MoO_x_ is reactive to water, leading to etching of (partially) oxidized MoS_2_ in water.^[^ ^197^, ^198^, ^199^ ^]^ Similarly, air exposure has shown to degrade the doping effect in the case for both stoichiometric and amorphous oxide dopants on TMDs.^[^ ^89^, ^200^ ^]^ Initial efforts have focused on using SiO_2_ or hBN encapsulation layers to improve the doping stability of oxides.^[^ ^128^, ^200^, ^201^ ^]^ In particular, Lee et al. demonstrated that the doping effect can be preserved over a year in oxidized WSe_2_ FETs.^[^ ^128^ ^]^ Thus, further developments of passivation techniques will be highly appealing to enhance the doping stability as well as thermal and chemical stability of oxidized TMDs.

To conclude, we have discussed the state‐of‐the‐art technologies for preparing oxidized TMDs along with their impact in a wide variety of application fronts – including atomic layer deposition, nanoelectronics, plasmonics, integrated photonics, and neuromorphic computing, just to name a few. Overall, the vast possibilities presented by oxidized TMDs suggest tremendous potential for growth and impact in fundamental studies for physics and chemistry as well as technology for industry‐ready applications for years to come.

## Conflict of Interest

The authors declare no conflict of interest.
